# Programmable synthetic receptors: the next-generation of cell and gene therapies

**DOI:** 10.1038/s41392-023-01680-5

**Published:** 2024-01-03

**Authors:** Fei Teng, Tongtong Cui, Li Zhou, Qingqin Gao, Qi Zhou, Wei Li

**Affiliations:** 1 University of Chinese Academy of Sciences, Beijing, 101408 China; 2 State Key Laboratory of Stem Cell and Regenerative Biology, Institute of Zoology, Chinese Academy of Sciences, Beijing, 100101 China; 3 Institute for Stem Cell and Regeneration, Chinese Academy of Sciences, Beijing, 100101 China; 4 Beijing Institute for Stem Cell and Regenerative Medicine, Beijing, 100101 China

**Keywords:** Biotechnology, Cell biology

## Abstract

Cell and gene therapies hold tremendous promise for treating a range of difficult-to-treat diseases. However, concerns over the safety and efficacy require to be further addressed in order to realize their full potential. Synthetic receptors, a synthetic biology tool that can precisely control the function of therapeutic cells and genetic modules, have been rapidly developed and applied as a powerful solution. Delicately designed and engineered, they can be applied to finetune the therapeutic activities, i.e., to regulate production of dosed, bioactive payloads by sensing and processing user-defined signals or biomarkers. This review provides an overview of diverse synthetic receptor systems being used to reprogram therapeutic cells and their wide applications in biomedical research. With a special focus on four synthetic receptor systems at the forefront, including chimeric antigen receptors (CARs) and synthetic Notch (synNotch) receptors, we address the generalized strategies to design, construct and improve synthetic receptors. Meanwhile, we also highlight the expanding landscape of therapeutic applications of the synthetic receptor systems as well as current challenges in their clinical translation.

## Introduction

As the next milestone in fighting diseases, cell and gene therapies are transforming the field of medicine by offering targeted and personalized treatments to patients that are not achievable by conventional pharmaceutics.^1–3^ As of now, chimeric antigen receptor T (CAR T) cell therapies for blood cancers,^4–6^ genetically engineered hematopoietic stem cells for hematologic disorders^7,8^ and gene therapies to treat a range of rare diseases including inherited retinal dystrophy and spinal muscular atrophy (SMA)^9–12^ are already clinically approved products on the market. The research and development continue to grow at a fast rate, with more novel therapies advancing in clinical development. However, moving to the next stage, there are major issues to be addressed. For cell and gene therapies, as the basic safety and efficacy feature, to precisely adjust the activity levels of the therapeutic cells or genes by controlling the active dosage, timing and localization is crucial.^13^ But currently, the overactivity of therapeutic cells and off-target effects in gene therapies are still significant obstacles to overcome. Uncontrolled CAR T cell activity can lead to the development of cytokine release syndrome (CRS) and neurotoxicity when infused CAR T cells become overactivated, causing severe or even life-threatening adverse events.^14,15^ While gene therapies involving introducing genetic materials into patients’ cells might disrupt the function or regulation of non-targeted genes, therefore causing serious consequences. As an intriguing and rapidly evolving field, synthetic biology is offering new solutions to address these problems.^13,16,17^ Novel synthetic receptor platforms are established as powerful tools to precisely control the function of engineered cells.^18,19^ They can be applied to finetune the therapeutic activities like adjusting production of dosed, bioactive payloads by sensing and processing user-defined signals or biomarkers.^13^ The convergence of synthetic biology with therapeutic strategies might substantially accelerate the evolvement of cell and gene therapies to the next generation.

Here, we review the current knowledge of synthetic receptor systems, including their characteristics and applications, as well as strategies to engineer and improve synthetic receptors. We also discuss the challenges for developing and adapting synthetic receptor platforms to program novel gene and cell therapies.

## Synthetic receptors: an overview

**Fig. 1:**
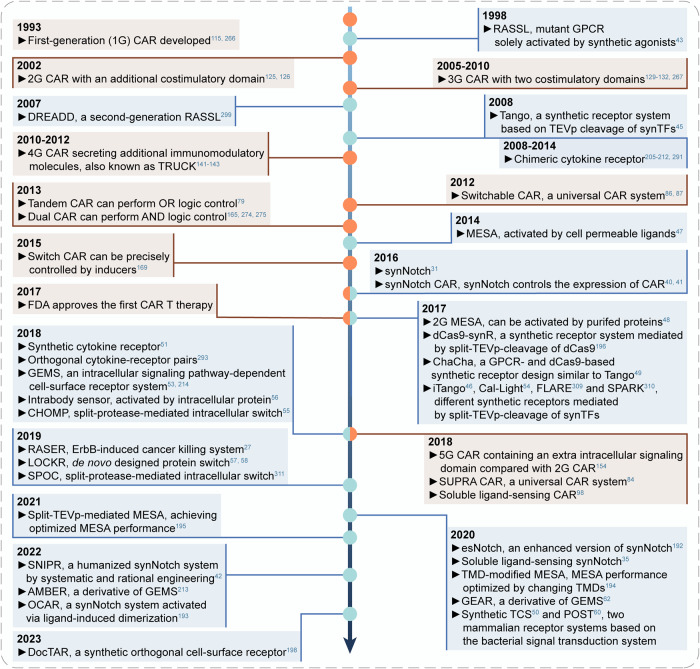
Landmark research achievements of the synthetic receptor over the past three decades. A timeline is shown with brief summaries of some of the key research milestones in the synthetic receptor field published in the past 30 years. CAR chimeric antigen receptor, RASSL receptor activated solely by a synthetic ligand, DREADD designer receptors exclusively activated by designer drug, TRUCK T-cells redirected towards universal cytokine killing, TEVp tobacco etch virus protease, synTF synthetic transcription factor, MESA modular extracellular sensor architecture, synNotch synthetic Notch, FDA the U.S. Food and Drug Administration, dCas9-synR dCas9 synthetic receptor, GPCR G protein-coupled receptor, iTango inducible Tango, Cal-Light calcium- and light-gated switch, FLARE fast light- and activity-regulated expression, SPARK specific protein associated tool giving transcriptional readout with rapid kinetics, SUPRA CAR split, universal and programmable CAR, RASER rewiring of aberrant signaling to effector release, LOCKR latching orthogonal cage-key protein, SPOC split-protease-cleavage orthogonal-coiled coil-based logic circuit, GEMS generalized extracellular molecule sensor, CHOMP circuits of hacked orthogonal modular protease, esNotch enhanced synNotch, TMD transmembrane domain, GEAR generalized engineered activation receptor, TCS two component system, POST phosphoregulated orthogonal signal transduction system, SNIPR synthetic intramembrane proteolysis receptor, AMBER advanced modular bispecific extracellular receptor, OCAR orthogonal chemically activated cell-surface receptor, DocTAR double-cut transcription activation receptor

Receptors empower cells to timely sense and respond to extrinsic (extracellular) and intrinsic (intracellular) stimuli in the complex environment. Based on the in-depth studies of various natural receptors over the past decades, synthetic biologists have been able to deconstruct and reconstruct receptors, and rationally engineer synthetic receptors. For a functional synthetic receptor, there are at least two domains: a sensor domain for specific binding with input signals, and an actuator domain to transduce sensor activity into outputs.^20^ Synthetic receptors can be engineered using natural components and/or artificial ones in origin,^20^ which endows cells (termed as ‘designer cells’) with customized functionalities by rewiring cellular input-output relationships.^20,21^ Figure 1 summarizes the timeline of key discoveries in synthetic receptor research.

**Fig. 2:**
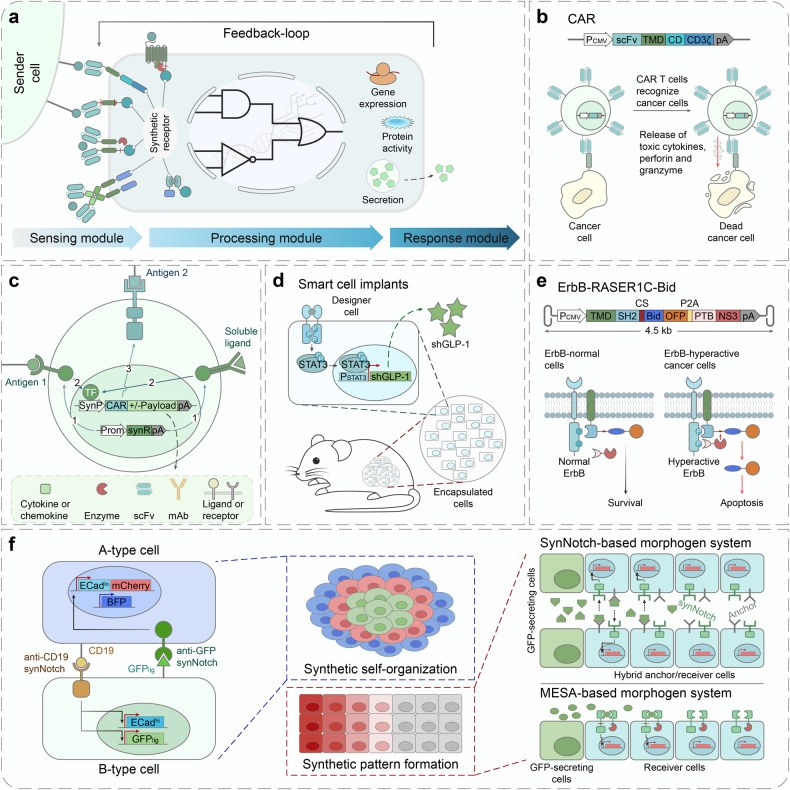
Programming cell and gene therapies using synthetic receptors. **a** Mammalian cells can be designed and engineered to sense and respond to a variety of stimuli such as chemicals and disease biomarkers, and subsequently trigger downstream signaling pathways, which can finetune customized therapeutic effects (e.g., gene expression, protein activity and secretion, etc.).^13,22^ **b** Engineering CAR T cell therapy.^68,96^ T cells are genetically engineered to express specific CAR proteins on their surface. When infused back into the body, CARs interact with the targeted antigens on cancer cells, causing the activation of CAR T cells for cancer-killing. P_CMV_ cytomegalovirus promoter, scFv single-chain fragment variant, TMD transmembrane domain, CD costimulatory domain, CD3ζ CD3 zeta signaling domain, pA poly(A) signal. **c** Synthetic receptor applications in CAR T cell therapy.^25,67^ T cells can be engineered with a combination of CARs and synthetic receptors like synNotch for a more precise tumor recognition to reduce off-target toxicity. Synthetic receptors like MESA could also be used in combination with CAR to sense a soluble biomarker. In addition to driving CAR expression, synthetic receptors are also able to express additional beneficial payloads alongside the CAR, such as cytokines, chemokines, enzymes, single-chain fragment variants (scFvs), mono-antibodies (mAbs), ligands or receptors. TF transcription factor, SynP synthetic promoter, Prom promoter, synR synthetic receptor, pA poly(A) signal. **d** Therapeutic cell engineering.^18,19^ By incorporating synthetic receptors, therapeutic cells are designed to act as ‘smart drugs’ that can sense disease biomarkers or user-defined inputs, and trigger a therapeutic response, such as the release of a drug or a therapeutic protein. These engineered cells present clinical potentials as they were encapsulated and implanted in mouse to treat diseases in proof-of-concept studies.^213,214^ STAT3, signal transducer and activator of transcription 3; P, phosphorylation; shGLP-1, synthetic human glucagon-like peptide 1. **e** Rewiring of aberrant signaling to effector release (RASER).^27,28^ In RASER, a hepatitis C virus protease (HCVp) and an effector protein (e.g., OFP-Bid) are fused to two different domains that can sense overactive ErbB signaling. When ErbB activity is detected, the two domains are co-recruited together, causing HCVp to cleave and activate OFP-Bid. This leads to the induction of apoptosis specifically in ErbB hyperactive cancer cells, sparing normal cells. The compact size of RASER construct makes it suitable for AAV-delivered gene therapy. P_CMV_ cytomegalovirus promoter, TMD transmembrane domain, SH2 Src homology 2 domain, CS cleavage site, Bid BH3 interacting domain death agonist, OFP orange fluorescent protein, P2A 2A peptide derived from porcine teschovirus-1, PTB phosphotyrosine-binding domain; NS3, hepatitis C virus nonstructural protein 3, pA poly(A) signal. **f** Engineering multicellular behaviors with synthetic receptor systems.^33,34^ (Left) To construct a three-layer structure, two separate cell lines are constructed using synNotch systems.^31,32^ CD19 ligands on the A-type cells can activate anti-CD19 synNotch receptors on the B-type cells, which induces the expression of Ecad^hi^ (E-cadherin, high expression) and GFP_lig_ (surface-bound GFP) in the B-type cells. Subsequently, these cells will form a two-layer structure with a green core and blue outer layer. Then, GFP_lig_ on the B-type cells can send reciprocal signals to the A-type cells via anti-GFP synNotch, leading to the activation of Ecad^lo^ (E-cadherin, low expression) and mCherry, which will induce the stepwise formation of the three-layer structure. (Right, Upper) Synthetic diffusive morphogen systems can be engineered using synNotch.^35^ In these systems, soluble ligands can form an artificial morphogen gradient and activate synthetic receptors on receiver cells. The gradient patterns can be tuned by modulating the expression level of synthetic morphogens (e.g., soluble GFP). The synNotch-based synthetic morphogen systems require an extra anchor protein to be expressed on the hybrid anchor/receiver cells (as shown here) or solely on the anchor cells. (Right, Lower) Another possible synthetic diffusive morphogen system using the synthetic receptor, such as MESA. In this supposed system, soluble ligands induce the dimerization of synthetic receptors, activating downstream gene transcription. GFP green fluorescent protein, mCherry a red fluorescent protein, BFP blue fluorescent protein, CD19 cluster of differentiation 19

Armed with synthetic receptors, designer cells are programmed to respond to multiple signals, achieving a spatiotemporal signaling and a subsequent behavior control^13^ (Fig. 2a). In a designer cell, there are three modules: the sensing module, the processing module and the response module.^13,22,23^ The sensing module includes but is not limited to various receptors that can detect a range of environmental cues, followed by signal transduction via downstream pathways.^13,22,23^ The processing module consists of rewired endogenous signaling pathways and orthogonal synthetic genetic circuits, which can process signals from multiple receptors.^13,22,23^ The response module are components generating measurable outputs, therefore employ user-defined changes (e.g., therapeutic effects)^13,22,23^ (Fig. 2a).

In recent years, the modular synthetic receptors have been constantly engineered and evolved for biomedical applications. As a well-known example, CARs are synthetic receptors to interact with target cells with high specificity. CAR T cell therapies (Fig. 2b) are now market-approved pharmaceuticals,^24^ which has convincingly demonstrated the great potential of synthetic receptors to be applied in therapeutics. Another widely used synthetic receptor is synthetic Notch (synNotch), which is able to conditionally drive the expression of the CAR as well as additional payload in engineered T cells when targeting a secondary antigen^25,26^ (Fig. 2c). Besides, synthetic receptors have been used to engineer therapeutic cells, which can sense arbitrary inputs, such as small molecules and disease-associated biomarkers, and secrete therapeutic molecules in response in animal models^19,23^ (Fig. 2d). Also, synthetic receptors with compact sizes (e.g., RASER (rewiring of aberrant signaling to effector release) as shown in Fig. 2e) can be directly delivered in targeted gene therapies by the AAV vector to treat ErbB-hyperactive cancers.^27,28^

Apart from biomedical applications, synthetic receptors have been widely adopted in the fundamental research.^29^ They are powerful tools to investigate various aspects of cell signaling and behaviors, including cell differentiation, migration and morphogenesis, in a controlled and precise manner.^30^ For example, researches have used synNotch receptors to program engineered-cells to self-organize into multicellular structures in response to juxtacrine signaling^31–34^ (Fig. 2f). Meanwhile, synthetic receptors can also be engineered as part of synthetic orthogonal morphogen systems to pattern three-dimensional (3D) tissues in a tightly controlled manner^34,35^ (Fig. 2f).

## Diverse types of synthetic receptors

Mammalian synthetic receptors can be classified based on different classification criteria. In this section, we provide an overview of diverse available synthetic receptors classified under four distinct principles. A summarization of the classified synthetic receptors is shown in Table 1, with their comparative advantages and limitations further discussed.

**Table 1:** Comprehensive overview of mammalian synthetic receptor systems

Type of synthetic receptor	Subtype of synthetic receptor	Type of ligand	Methodology	Performance	Comments	References
Chimeric antigen receptor (CAR)	1 G CAR	Surface-bound ligand	Activate similar endogenous TCR pathways by immunoreceptor tyrosine-based activation motifs in the ICD	(+) Engineered T cell therapy (+) Highly programmable (−) Low cell proliferation and short lifespan in vivo (−) Lack of efficacy	● CARs are fully humanized synthetic receptors, with minimal immunogenicity per se ● Six CAR T cell therapies based on 2 G CARs have been approved by the FDA ● Despite the clinical efficacy proved in blood cancer, serious complications can occur, including CRS, “on-target, off-tumor toxicity”, etc. ● The lymphodepleting chemotherapy administered before CAR T cell infusion is genotoxic ● CAR T cell therapy for solid tumors is challenging largely due to antigen heterogenecity, tumor microenvironment, and inefficient T cell trafficking and infiltration ● Next-generation CARs are developed to enhance T cell persistence and cytotoxicity, thus improving antitumor activity ● Future trials are needed to investigate the safety and efficacy of newly developed CARs ● CAR T cell therapy hold promise for treating diseases other than cancer ● The cost of engineering and producing CAR T cells is high In vivo CAR T platforms have a huge potential to reduce manufacturing costs and obviate the need for lymphodepletion, though less mature	Res.^115,259^ Rev.^39,63,99,101,114^
	2 G CAR		Comprise one additional costimulatory domain compared to 1 G CARs	(+) Enhance T cell proliferation and cytotoxicity (+) Increase cytokine production (−) Might possess constitutive or tonic activity		Res.^125,126^ Rev.^39,63,99,101,114^
	3 G CAR		Contain two additional costimulatory signaling domains in *cis* compared to 1 G CARs	(+) Increased persistence and proliferation of CAR T cells (−) No enhanced efficacy compared to 2 G CAR T cells		Res.^129–132,260^ Rev.^39,63,99,101,114^
	4 G CAR (T-cells redirected towards universal cytokine killing (TRUCK))		Constitutively or inducibly secrete immunomodulatory molecules, including cytokines, antibodies, enzymes, etc. compared to 2 G CARs	(+) Promote tumor killing through synergistic mechanisms (+) Avoid systemic toxicity (−) Supra-therapeutic level of cytokines might be produced and limit therapeutic efficacy (−) Off-target cytokine delivery may produce side effects		Res.^141–150^ Rev.^99,114,151,152^
	5 G CAR		Contain an extra distinct intracellular signaling domain than 2 G CARs	(+) Induce endogenous cytokine signaling without cytokine secretion (+) Enhance antitumor effects of CAR T cells (+) Enhance synapse formation and CAR NK cell polarization (−) Potentially increase the risk for cytokine releasing syndrome (CRS) due to CAR T cell persistence		Res.^154,261^ Rev.^114^
	Tandem CAR		Bispecific (or multiple) antigen binders in the ECD	(+) Perform OR logic gate control of T cell responses (+) Effective against heterogeneous tumors (+) Achieve synergistic antitumor efficacy (+) Prevent antigen escape	● AND logic gate can improve targeting specificity whereas OR gate can attenuate antigen escape ● Tandem linked scFvs should be carefully optimized to reserve their function Multi-antigen targeting using OR logic strategies may increase the risks associated with CRS and on-target, off-tumor toxicity	Res.^79–81,262–267^ Rev.^20,68,99,101,263^
	Dual CAR		Two different CARs expressed in on cell	(+) Perform AND logic gate control by expressing two complementary CARs (+) Perform OR logic gate control by expressing two intact CARs	● Remote and spatiotemporal control of CAR T cells could improve the safety and efficacy of CAR T cell therapy ● CAR activity can be reversibly modulated, thus preventing CRS or on-target, off-tumor toxicity Additional modules are incoporated into CAR constructs, and a cognate inducer is required. These should be both carefully evaluated in translational studies	Res.^165,166,268–272^ Rev.^20,68,99,263,273–275^
	Switch CAR^a^		Small molecules/physical stimulations control CAR activity via inducible assembly or stabilization	(+) CAR activity can be precisely spatiotemporally controlled (+) Perform safety control (+) Prevent on-target off-tumor toxicity		Res.^169,171,172,220,276–281^ Rev.^68,97,101,273,274^
	Switchable CAR^b^		Bispecific adaptors redirect a universal CAR to different antigens	(+) Possess an intrinsic switch for safety control (+) Versatile for specificity programming	● Highly versatile, easily adjustable and programmable ● May significantly reduce the cost of CAR T cell therapy ● Require repeated administration of bispecific adaptors ● The safety and efficacy of adaptors per se should be validated in clinical studies The UniCAR designs can be engineered into allogenic “off-the-shelf” T cells	Res.^86–95,282^ Rev.^99^
	Split, universal, and programmable (SUPRA) CAR^b^		Reconstruct a split CAR into an intact protein using leucine zipper pairs	(+) Enhance programmability of CARs (+) Fine-tune CAR activation to mitigate toxicity (+) Perform Boolean logic computation in CAR T cells		Res.^84,85^ Rev.^274,283^
	Soluble ligand-sensing CAR	Extracellular soluble ligand	Induce dimerization of CARs by soluble ligands	(+) First demonstrate CARs can be engineered to respond to soluble ligands (−) The therapeutic use needs further exploitation	● CAR T cells can be engineered to respond robustly to a variety of soluble ligands ● On-target, off-tumor toxicity should be noted with caution, since CAR T cells might be pre-activated by systemic disease biomarkers	Res.^98^
Chimeric cytokine receptor	scFv-EpoR_D1D2_-cytokine receptor chimera^c^	Extracellular soluble ligand	Induce chimeric cytokine signal using antibody fused EpoR_D1D2_ scaffold	(+) Highly programmable (+) Perform a strict ligand-dependent ON/OFF switch (−) Respond to native ligand	● Chimeric cytokine receptors are able to mimic the function of natural cytokine receptors ● Chimeric cytokine receptors can sustain CAR T cell function using a non-native ligand instead of the cytokine, thus avoiding potential adverse consequences led by cytokine pleiotropy ● Require repeated injection of the inducer or co-expression of a protein inducer, whose safety (e.g., potential toxicity) should be evaluated The chimeric receptor construct can be humanized to mitigate potential immunogenicity	Res.^205,206^
	scFv-EpoR_D2_-cytokine receptor chimera	Extracellular soluble ligand	Induce chimeric cytokine signal using antibody fused EpoR_D2_ scaffold	(+) Highly programmable (+) Efficiently induce cytokine signal in the presence of non-natural ligands (+) Don’t respond to native ligand		Res.^207,210–212^ Rev.^284^
	CID-cytokine receptor chimera		Functionalized chimeric cytokine receptors using chemically induced dimerization (CID)	(+) Highly programmable (+) Engineer functional unnatural heterodimeric receptors (+) Reduce off-target effects (−) Potential toxicity of chemical small molecules		Res.^214,285^ Rev.^284^
	Synthetic cytokine receptor (SyCyR)		Chimeric cytokine receptors	(+) Highly programmable (+) Induce cytokine signaling using non-natural ligands (+) Easily switch on/off by soluble nanobodies		Res.^51^ Rev.^20,286^
	Orthogonal cytokine-receptor pairs		Engineer mutant cytokine and mutant cognate receptor (orthogonal pairs)	(+) Enable selective cytokine signal (+) Reduce off-target effects (+) With negligible systemic toxicity		.Res.^287–290^ Rev^286,291^
Generalized extracellular molecule sensor (GEMS)	Generalized extracellular molecule sensor (GEMS)^c^	Extracellular soluble ligand	Program user-defined input-output relationships by rewiring endogenous pathways	(+) Highly programmable (+) Intrinsic cascade amplification provides a high signal-to-noise ration (+) Detect pathological concentrations of disease biomarkers (−) Disturb endogenous signaling pathways	● GEMS shows promise in treating diseases via engineered cells in preclinical models ● GEMS (like TRUCK) can use natural signaling to modulate cell function and induce additional expression of transgenes, which brings clinical benefits ● GEMS can be fully humanized to mitigate immunogenicity GEMS-engineered cells meet big challenges in translational research, since endogenous signaling activation might bring tumorigenic risks	Res.^53,214^ Rev.^18,20^
	Generalized engineered activation regulator (GEAR)		Rewire endogenous pathways to genomic targets using engineered activation regulators based on GEMS	(+) Highly programmable (+) High sensitivity (+) High fold induction (+) Activate native genomic gene expression (−) Disturb endogenous signaling pathways		Res.^62^ Rev.^20^
	Advanced modular bispecific extracellular receptor (AMBER)		DARPin-based GEMS receptors	(+) Highly programmable (+) Enable high-throughput screening (+) Detect pathological concentrations of disease biomarkers (−) Disturb endogenous signaling pathways		Res.^213^
Receptor activated solely by a synthetic ligand (RASSL)	1 G RASSL	Extracellular soluble ligand	Site mutation of G protein-coupled receptors (GPCRs) to respond to synthetic agonists while unresponsive to endogenous ligands	(+) Receptors activated by synthetic ligands (−) Off-target effects of synthetic ligands (−) High constitutive activity (−) Limited programmability	● A powerful chemogenetic technology for remote control over neuronal activity and cellular signaling ● Show potential applications in treating diseases like eating disorders, obesity and obesity-associated metabolic abnormalities ● The outcomes achieved by DREADD activation are cell-type specific In vivo expression of cell-type specific DREADD is challenging due to the difficulties in delivery, which renders its application in gene therapy	Res.^43,292^ Rev.^44,67^
	2 G DREADD (designer receptors exclusively activated by designer drug)		Directed evolution of GPCRs	(+) Show insensitivity to endogenous ligands (+) Have low constitutive activity (+) Fewer off-target effects if any (+) Most widely employed chemogenetic tools (−) Limited to GPCRs (−) Limited programmability		Res.^218,293–296^ Rev.^44,67,297–299^
Synthetic Notch (synNotch)	synNotch	Surface-bound ligand	Rational design of modular chimeric receptor using mouse Notch regulatory region	(+) Highly programmable (+) Orthogonal signal transduction (+) Can enhance the specificity and sensitivity of CAR T cells (+) Enable orthogonal cell-cell communication (−) A high level of background activity	● Highly robust ● Widely used in CAR T cell engineering to enhance the function of CAR T cells in various aspects ● Show promise in translational and clinical applications by means of adoptive cellular therapy ● Fully humanized Not suitable for gene therapy via AAV delivery due to the relatively large size	Res.^31,32,40,41,167,168,189,190,252,300^ Rev.^18,20,26,33,34,67,101,273^
	Enhanced synNotch (esNotch)		Add a native Notch-derived intracellular hydrophobic sequence (QHGQLWF) into synNotch receptors	(+) Highly programmable (+) Orthogonal signal transduction (+) Significantly reduced ligand-independent activation		Res.^192^ Rev.^20^
	Synthetic intramembrane proteolysis receptor (SNIPR)		Systematic and rational engineering of synNotch receptors	(+) Highly programmable (+) Orthogonal signal transduction (+) Minimal size for easier cell engineering (+) Humanization with low immunogenicity (+) Program CAR T cell engineering		Res.^42^ Rev.^20^
	Soluble ligand-sensing synNotch	Extracellular soluble ligand (anchored)	Fix soluble ligands onto cell surface by anchor protein	(+) Highly programmable (+) Perform orthogonal morphogen signaling (−) Require expression of an additional anchor protein	● Facilitate the basic research by engineering synthetic systems Can hardly be utilized in translational research	Res.^35^ Rev.^34^
	Orthogonal chemically activated cell-surface receptor (OCAR)	Extracellular soluble ligand and surface-bound ligand	Chemically induce dimerization of repurposed synNotch receptors	(+) Highly programmable (+) Orthogonal signal transduction (+) Possess an intrinsic off-switch control (+) Inducibly increase synNotch activity during cell-cell communication	● More complicated than synNotch systems ● Can synergize with synNotch to function, but as an off-switch, OCAR cannot completely shut down the activity of co-expressed synNotch receptor when sender cells present	Res.^193^
TEVp-based sensor	Modular extracellular sensor architecture (MESA)	Extracellular soluble ligand	Modular synthetic receptors function via induced heterodimerization	(+) Highly programmable (+) Orthogonal signal transduction (−) Require extensive tuning of the expression of the target and protease chains (−) High background activity and low induced fold change (−) Require high concentration of inducers	● Proposed to be able to sense various systemic disease biomarkers in potential applications ● Might be humanized in the future High background signal and low signal-to-noise ratio hinder its wide application	Res.^47,48^ Rev.^18,20,67,298^
	TMD-modified MESA		Refined MESA receptors via optimizing TMDs	(+) Highly programmable (+) Orthogonal signal transduction (+) Improved performance (−) Require extensive tuning of the expression of the target and protease chains (−) Require high concentration of inducers		Res.^194^ Rev.^20^
	Split-TEVp-mediated MESA		Computation-guided workflow to rationally design split-TEVp for improving MESA	(+) Highly programmable (+) Orthogonal signal transduction (+) Computational design and modeling (+) Achieve low background and high fold induction (−) Require high concentration of inducers		Res.^195^ Rev.^20^
	dCas9 synthetic receptor (dCas9-synR)		Split-TEVp-mediated cleavage of split-dCas9 from chimeric receptors via induced proximity	(+) Highly programmable (+) Orthogonal signal transduction (+) Activate native genomic gene expression (+) Integration of user-defined AND logic gate (−) Natural inputs (−) Require high concentration of inducers		Res.^196^ Rev.^20^
	Tango	Extracellular / intracellular soluble ligand	TEVp-based cleavage of synTFs from chimeric receptors via induced proximity	(+) Highly programmable (+) Achieve orthogonal output function (−) High background signal and poor signal-to-noise ration (−) Required arrestin / PTB recruitment (−) Disturb endogenous signaling pathways	● Originally designed as a reporter system to monitor protein interaction, receptor activation or neural activity ● The incorporation of light-sensitive domain improves the sensitivity and specificity of these synthetic receptors ● Can be used to facilitate drug screening ● Can be repurposed to control therapeutic gene expression	Res.^45,301,302^ Rev.^18,20,28,67^
	iTango/iTango2 (inducible Tango)	Extracellular soluble ligand and light	Ligand- and light-dependent split-TEVp cleavage of synTFs via induced proximity	(+) Highly programmable (+) Achieve orthogonal output function (+) Possess an external control switch (+) Perform highly spatiotemporal control (−) Required arrestin recruitment (−) Disturb endogenous signaling pathways		Res.^46^ Rev.^28^
	Calcium- and light-gated switch (Cal-Light)	Ca^2+^ and light	Neuronal-activity-mediated calcium signaling and light-dependent split-TEVp cleavage of synTFs via induced proximity	(+) Highly programmable (+) Achieve orthogonal output function (+) Possess an external control switch (+) Perform highly spatiotemporal control (−) Restrict to increased cytosolic Ca^2+^ levels triggered by neuronal activity (−) Disturb endogenous signaling pathways		Res.^54^ Rev.^20^
	Fast light- and activity-regulated expression (FLARE)		Neuronal-activity-mediated calcium signaling and light-dependent TEVp cleavage of synTFs via induced proximity	(+) Highly programmable (+) Achieve orthogonal output function (+) Possess an external control switch (+) Perform highly spatiotemporal control (−) Restrict to increased cytosolic Ca^2+^ levels triggered by neuronal activity (−) Disturb endogenous signaling pathways		Res.^303^ Rev.^28^
	Specific protein associated tool giving transcriptional readout with rapid kinetics (SPARK)	Extracellular soluble ligand and light	Ligand- and light-dependent TEVp cleavage of synTFs via induced proximity	(+) Highly programmable (+) Achieve orthogonal output function (+) Possess an external control switch (+) Perform highly spatiotemporal control (−) Required arrestin recruitment (−) Disturb endogenous signaling pathways		Res.^304^ Rev.^28^
	ChaCha	Extracellular soluble ligand	TEVp-based cleavage of synTFs from chimeric GPCRs via induced proximity	(+) Highly programmable (+) Outperform the Tango design (+) Activate native genomic gene expression (−) Required arrestin recruitment (−) Disturb endogenous signaling pathways	● An evolved variant of Tango that can drive endogenous gene expression via dCas9-TFs Might be applied in cell engineering whereas not suitable for gene therapy	Res.^49^ Rev.^20,28^
	Rewiring of aberrant signaling to effector release (RASER)	Active ErbB^d^	Protease cleavage release effectors via phosphorylation induced proximity	(+) Highly programmable (+) High specificity and efficacy (+) Serve as an example of the ability of mathematical models (+) Compact size suitable for AAV delivery (−) Required PTB recruitment	● Hyperactivity of ErbB triggers the activation of RASER, releasing a programmable effector to control gene expression or cell death ● Can be used for cancer gene therapy, but in vivo experiments are lacking	Res.^27^ Rev.^20,197^
	Intrabody sensor	Intracellular soluble ligand	TEVp-mediated cleavage release synTFs via intracellular nanobody-mediated induced proximity	(+) Highly programmable (+) Orthogonal signal transduction (−) Require two nanobodies that bind two distinct epitopes of the target protein	● Intracellular disease biomarkers trigger its activation ● Lack in vivo assessment of therapeutic potential ● Require in vivo delivery	Res.^56^ Rev.^20,28,197^
	Circuits of hacked orthogonal modular protease (CHOMP)		Split protease-mediated cleavage release effectors via dimerization	(+) Highly programmable (+) Implement binary logic gates	● Can be engineered as a switch to control gene expression	Res.^55^ Rev.^20,28,197^
	Split-protease-cleavage orthogonal-coiled coil-based (SPOC) logic circuit		Split protease-mediated cleavage release effectors via dimerization	(+) Highly programmable (+) Implement binary logic gates (+) De novo design coiled coils		Res.^305^ Rev.^20,28,197^
Mammalian-membrane two-hybrid (MaMTH) assay		Soluble ligand	Ligand- and phosphorylation-dependent split-ubiquitin cleavage of synTFs via induced proximity	(+) Highly programmable (+) Achieve orthogonal output function (+) Perform high-throughput drug screening (−) Required arrestin / PTB recruitment	● Designed as a reporter system to monitor protein interaction ● Has facilitated drug discovery ● Can be repurposed to control therapeutic gene expression	Res.^306–309^ Rev.^28^
Latching orthogonal cage-key protein (LOCKR)		Intracellular soluble ligand/surface-bound ligand	The functional peptide embedded in the latch can function only when the key displaces the latch from the cage	(+) Highly programmable (+) De novo designed protein switches (+) Rational computational design (+) Perform logic gate control	● De novo designed protein switch can function in cells ● Colocalization-dependent LOCKR (Co-LOCKR) system can display a logic computation capability based on a UniCAR design ● Further improvement is needed in translational applications, especially for the NOT logic circuit The pharmacokinetics and immunogenicity need to be further assessed	Res.^57,58,94,310^ Rev.^97,197,311^
Synthetic Ca^2+^ actuator	Chimeric VEGFR2	Extracellular soluble ligand	Induced dimerization if VEGFR2 generates a Ca^2+^ signal	(+) Highly programmable (+) Rewire a wide range of extracellular stimuli to intracellular Ca^2+^ signal (−) Activate other endogenous signaling pathways	● Through modulating Ca^2+^ signaling, chimeric VEGFR is able to program cells to migrate toward a site where an extracellular ligand is expressed Its potential to trigger other signaling pathways should be noted	Res.^312,313^ Rev.^20^
	Light-inducible membrane-tethered peripheral ER (LiMETER)/optoPBer	Light	Light induces ER-tethered LOV2-PB protein to restore its PI-interacting capability, and open ORAI channel to trigger Ca^2+^ influx	(+) Enable real-time photo-inducible ER-PM membrane contact site (MCS) assembly (+) Quantitatively and qualitatively control intracellular Ca^2+^ levels (−) Require addition expression of ORAI	● Enables Ca^2+^ signaling activation in both excitable and non-excitable cells ● Opto-CRAC can induce activation of the olfactory sensory neuron and trigger electro-olfactogram responses in mice ● OptoSTIM1 expressed in CA1 hippocampus can modulate the emotion circuits and enhance the learning capacity of mammals LOCa can modulate abberant self-renewal of hematopoietic stem cells, and mitigate neurodegeneration in a *Drosophila* model of Alzheimer’s disease Challenges remain in their in vivo delivery and optogenetic approaches	Res.^314,315^ Rev.^316–319^
	LOV2-STIM1ct (Blue light-activated Ca^2+^ channel switch (BACCS)/opto-CRAC)	Light	Light induces activation of chimeric LOV2-STIM1ct, opening ORAI channel to trigger Ca^2+^ influx	(+) Selectively and remotely control Ca^2+^ signal (+) Regulate the function of non-excitable cells (+) Enable orthogonal output function (−) Require addition expression of ORAI		Res.^320–324^ Rev.^317–319^
	CRY2-STIM1ct (optoSTIM1)	Light	Light induces oligomerization of CRY2-STIM1ct, opening ORAI channel to trigger Ca^2+^ influx	(+) Selectively and remotely control Ca^2+^ signal (+) Regulate the function of non-excitable cells (+) Enable orthogonal output function (−) Require addition expression of ORAI		Res.^324,325^ Rev.^317–319^
	COSMO-STIM1ct	Intracellular soluble ligand	Caffeine induces oligomerization of COSMO-STIM1ct, opening ORAI channel to trigger Ca^2+^ influx	(+) Selectively and remotely control Ca^2+^ signal (+) Regulate the function of non-excitable cells (+) Enable orthogonal output function (−) Require addition expression of ORAI		Res.^326^ Rev.^317–319^
	optoRGK	Light	Light induces cytosol-to-plasma membrane translocation of engineered RGK GTPases and modulates Ca_V_ channel activity	(+) Spatiotemporal control of Ca^2+^ signal (−) The efficacy of optoRGK highly depends on Ca_V_ abundancy		Res.^327^ Rev.^317–319^
	Light-operated Ca^2+^ (LOCa) channel	Light	Light induces engineered ORAI1 activation by inserting a photosensory LOV2 into its intracellular loop	(+) Precisely and reversibly control Ca^2+^ signals (+) A single component and compact size, suitable for gene delivery		Res.^328^ Rev.^317–319^
Two component system (TCS)	Synthetic TCS	Extracellular/intracellular soluble ligand	Induce dimerization of prokaryotic two-component systems in mammalian cells	(+) Highly programmable (+) Orthogonal signal transduction (+) Dose-dependent signaling (−) Bacterial origin of TCS	● Orthogonal signaling system ● TCS is derived from the bacteria, so might elicit immunogenicity in clinical applications ● Inducers can be human-friendly	Res.^50^ Rev.^197^
	Phosphoregulated orthogonal signal transduction (POST) system	Intracellular soluble ligand	Induce dimerization of prokaryotic two-component systems in mammalian cells	(+) Highly programmable (+) Orthogonal signal transduction (+) Dose-dependent signaling (−) Bacterial origin of TCS		Res. ^60^ Rev. ^20,197^

^a^Here we narrowly describe switch CAR systems built via solely engineered CAR architecture. Broadly speaking, switch CAR systems can also be constructed via induced transcription expression for CAR expression, like synNotch CAR and TetOn-induced CAR^68,101,275,329^

^b^Here we describe switchable CAR systems as a unique CAR T cell can be redirected to target a new antigen by adding a bispecific adaptor protein. And in this sense, SUPRA CARs also belong to these systems

^c^The ScFv-EpoR_D1D2_-cytokine receptor chimera should be considered as the prototype of the GEMS platform

^d^Constitutively active ErbB rather than transiently activated ErbB induces RASER proteins to release a programmable effector

+ and − represent advantages and disadvantages, respectively. Res. and Rev. represent research articles and review articles, respectively

*ICD* intracellular domain, *ECD* extracellular domain, *TMD* transmembrane domain, *1* *G* first-generation, *DARPin* designed ankyrin repeat protein, *TEVp* tobacco etch virus protease, *synTF* synthetic transcription factor, *ER* endoplasmic reticulum, *LOV* light, oxygen, or voltage domain, *PB* polybasic domain, *ORAI* ORAI calcium release-activated calcium modulator, *STIM1* stromal interaction molecule 1, *STIM1ct* STIM1 cytoplasmic domain, *VEGFR2* vascular endothelial growth factor receptor 2, *CRY2* cryptochrome circadian regulator 2, *RGK GTPases* Ras-like GTPases Rad/Rem/Gem/Kir, *COSMO* caffeine-operated synthetic module

### Cell-surface receptors and intracellular receptors

According to the location to bind ligands, synthetic receptors can be divided into cell-surface receptors (also known as transmembrane receptors) and intracellular receptors.^36^

Cell-surface receptors generally comprise three types of transmembrane receptors (i.e., enzyme-linked receptors, G-protein-coupled receptors and ion channel-liked receptors), which span the plasma membrane and convert extracellular signals into intracellular signals.^37,38^ For this category, each synthetic receptor contains an extracellular ligand-binding domain, at least one transmembrane domain and an intracellular effector domain. Besides the best-known CAR,^24,39^ synNotch,^31,40,41^ SNIPR (synthetic intramembrane proteolysis receptor),^42^ RASSL (receptor activated solely by a synthetic ligand),^43,44^ Tango,^45,46^ MESA (modular extracellular sensor architecture),^47,48^ ChaCha,^49^ TCS (two-component system),^50^ chimeric cytokine receptor^51,52^ and GEMS (generalized extracellular molecule sensor)^53^ also fall into the category of cell-surface receptors (Table 1). This type of synthetic receptors can only sense external signals, making them suitable to detect cellular (e.g. CAR, synNotch and SNIPR) and systemic disease biomarkers (e.g. MESA and GMES).

Intracellular receptors can either locate in the cytoplasm or nucleus, or be anchored to the intracellular membrane of the cell. Cal-Light (calcium- and light-gated switch),^54^ CHOMP (circuits of hacked orthogonal modular protease),^55^ intrabody sensor,^56^ RASER (rewiring of aberrant signaling to effector release),^27^ LOCKR (latching orthogonal cage-key proteins),^57,58^ COMET (composable mammalian elements of transcription)^59^ and POST (phosphoregulated orthogonal signal transduction)^60^ belong to the category of intracellular receptors (Table 1). Notably, the synthetic receptors discussed here could also be referred to as synthetic protein switches.^61^ Since intracellular receptors can only be activated by intracellular input, many of them (e.g., LOCKR and COMET) are designed as a switch induced by chemical molecules that can cross the plasma membrane.

### Natural signaling-based receptors and orthogonal signaling-based receptors

Activated receptors trigger signal transduction via multiple downstream pathways. According to the downstream pathway actuated, either natural or engineered, we divide synthetic receptors into natural signaling-based receptors and orthogonal signaling-based receptors.

Natural signaling-based receptors rewire endogenous signaling pathways to either original or customized outputs. This category of synthetic receptors includes RASSL,^43,44^ CAR,^24,39^ chimeric cytokine receptor,^51,52^ GEMS^53^ and GEAR^62^ (Table 1). They inevitably activate native pathways to actuate outputs. Among them, RASSL, CAR and chimeric cytokine receptor redirect endogenous pathways to user-defined ligands to execute desirable functions, and are no longer responsive to their original ligands.^18–20^ While GEMS and GEAR hijack endogenous pathways to generate additional customized outputs, they can simultaneously lead to activation of endogenous transcription networks.^18–20^ On the other hand, the capacity to modulate transcriptional networks endows natural signaling-based receptors with unique advantages over orthogonal signaling-based receptors, allowing for the execution of natural and highly complex programs. For example, CARs are invented to mimic the function of T-cell receptor (TCR) to achieve antitumor activity^63^ via modulating endogenous signaling pathways, but at the same time, additional transgenes can be induced by CAR activation (e.g., nuclear factor of activated T cells (NFAT)-driven cytokine expression), which could not be achieved by orthogonal systems.

By contrast, orthogonal signaling-based receptors are completely independent of endogenous signaling pathways. This attributes to the employment of orthogonal synthetic transcription factors (syn-TFs) or orthogonal signal transduction systems, as they cannot recognize endogenous regulatory elements and activate endogenous signaling cascades, respectively.^18–20^ Nevertheless, some syn-TFs like dCas9-based TFs and TALE-based TFs can program endogenous gene expression without the requirement of extra synthetic promoters.^18,20^ The currently available Tango,^45,46^ MESA,^47,48^ synNotch,^31,40,41^ ChaCha,^49^ Cal-Light,^54^ intrabody sensor,^56^ LOCKR^57,58^ and COMET^59^ employ syn-TFs for orthogonal signal pathway construction, while TCS^50^ and POST^60^ utilize orthogonal signaling systems derived from prokaryotes (Table 1).

Of note, the most widely used orthogonal TFs are of non-human origin (e.g., yeast and bacteria), therefore carrying high risks of immunogenicity in clinical applications. The use of humanized syn-TFs comprising both the DNA-binding domain and activation domain derived from human TFs will largely reduce the immunogenic risk. However, these fused TFs are still able to activate endogenous signaling, which reduces their orthogonality. Notably, Khalil and colleagues de novo designed a panel of fully humanized synthetic zinc finger transcription regulators (synZiFTRs) engineered by an array of zinc finger domains, which can specifically recognize their cognate short DNA-binding motifs, achieving genome-orthogonal specificities.^64^ This kind of pioneering work will accelerate the progress of humanization of orthogonal signaling-based receptors, which is pivotal to bridge the translational gap to the clinic.

### Soluble ligand-binding receptors and surface ligand-binding receptors

Synthetic receptors can bind to a range of soluble and surface-bound ligands with a high specificity and sensitivity. In line with the features of ligands, the corresponding synthetic receptors can be categorized into soluble ligand-binding receptors and surface ligand-binding receptors.^18,21^

A large variety of ligands are in the soluble form, including most chemical molecules, hormones, cytokines, growth factors, intracellular soluble proteins, and some peptides cleaved from membrane proteins (e.g., carcinoembryonic antigen (CEA)^65^ and amyloid-beta (Aβ)^66^). The above listed soluble molecules can induce the activation of soluble ligand binding-receptors, such as Tango,^45,46^ ChaCha,^49^ MESA,^47,48^ chimeric cytokine receptor,^51,52^ CHOMP,^55^ GEMS^53^ LOCKR^57,58^ and POST^60^ (Table 1). By using small chemical molecules that can cross the plasma membrane, several intracellular synthetic receptors (e.g., POST, CHOMP and LOCKR) have been developed.^20^ Meanwhile, they can also act as extracellular ligands when the ligand-binding domain of a receptor locates outside the plasma membrane, *scilicet* cell-surface receptors (e.g., Tango, ChaCha, MESA and GEMS).^20^ For peptide/protein ligands, in most cases, they cannot enter the cell, and therefore act on cell-surface receptors to subsequently trigger downstream intracellular signaling cascades.

Surface-bound ligands fixed on the plasma membrane of sender cells can *trans*-activate cell-surface receptors on adjacent receiver cells.^18,20^ CAR and synNotch are typical surface ligand-sensing receptors which theoretically cannot be activated by soluble ligands.^18,20^ The different properties of these two types of synthetic receptors possess distinct mechanisms of action. Soluble ligand-binding receptors are usually activated by ligand-induced dimerization to trigger downstream signaling, whereas surface ligand-binding receptors require a mechanical force generated by ligand-receptor interaction to activate downstream signaling. Therefore, activation of soluble ligand-binding receptors (e.g., chimeric cytokine receptor and GEMS) can be induced by ligands after a long-distance transport, whereas surface ligand-binding receptors (CAR and synNotch) can only function in a juxtacrine manner.^34^

### Partially modular receptors and fully modular receptors

Over the past decade, an increasing number of synthetic receptor systems have been engineered and refined. Based on whether they are made up entirely of reconfigurable components, we divide synthetic receptors into partially modular receptors and fully modular receptors.

Partially modular receptors can be constructed by engineering the artificial sensor domain while retaining the original actuator domain, or vice versa. The former synthetic receptors can rewire endogenous signaling pathways to new ligands (e.g., CAR, chimeric cytokine receptor and GEMS), while the latter ones can activate an alternative pathway by natural ligands (e.g., Tango, ChaCha and dCas-synR)^18–20^ (Table 1). Therefore, the former synthetic receptors can be categorized as natural signaling-based receptors and the latter ones as orthogonal signaling-based receptors (as discussed above).

Fully modular synthetic receptors, with both the sensor domain and actuator domain engineered, including synNotch,^31,40,41^ SNIPRs,^42^ MESA^47,48^ and LOCKR,^57,58^ can execute novel functions without disrupting the endogenous pathways in an orthogonal way (Table 1). It is worth noting that the ‘building brick’ for modular assembly of fully modular synthetic receptors can be either derived from either pre-exiting natural components (e.g., synNotch, SNIPRs and MESA) or de novo designed ones (e.g., LOCKR).^20^

## Engineering of synthetic receptors

**Fig. 3:**
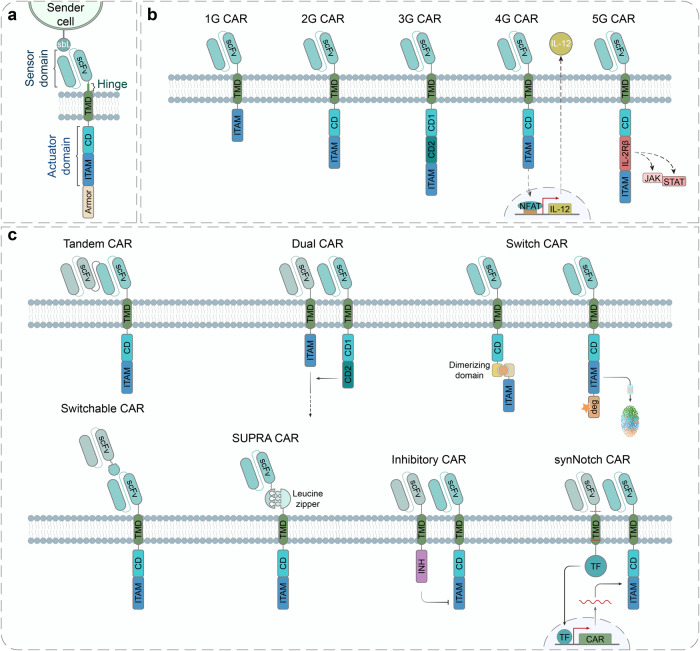
Design and engineering of the chimeric antigen receptor (CAR). **a** The architecture of CARs comprises an extracellular sensor domain, a hinge, a transmembrane domain (TMD) and an intracellular signaling domain (actuator domain).^330^ The extracellular sensor domain, also known as antigen-binding domain, is usually a single-chain variable fragment (scFv) derived from a monoclonal antibody by fusing its light (V_L_) and heavy (V_H_)-chain variable domain with a flexible linker peptide. Other proteins like nanobodies, designed ankyrin repeat proteins (DARPins), natural ligands and small peptides can also function as the antigen-targeting moiety.^99,101^ The hinge derived from T cell proteins or immunoglobins can function as a flexible linker, providing sufficient conformational freedom to overcome steric hinderance to facilitate the access to the target antigen. T cell protein-derived or de novo designed TMD not only anchors the CAR in the cell membrane but also affects the stability and function of CAR. The intracellular signaling domain generally contains a CD3ζ signaling domain and CD28/4-1BB costimulatory domains (CDs), which facilitates T cell persistence and activity. Several other costimulatory domains including ICOS, OX-40, CD27, MyD88/CD40 and NKG2D are already underway.^99,101^ CAR architectures can be further engineered to express an ‘armor’, which aims to enhance the in vivo persistence and efficacy of CAR T cells. sbL, surface-bound ligand; ITAM, immunoreceptor tyrosine-based activation motif. **b** First-generation (1 G) CARs only contain a CD3ζ signaling domain in the intracellular domain (ICD), which outperforms the less popular FcεR1γ signaling domain. Second-generation (2 G) CARs harbor one CD, and third-generation (3 G) CARs contain more than one CDs in their intracellular signaling domain. Fourth-generation (4 G) CARs are based on 2 G CARs with additional expression of transgenic products (armor), such as cytokines, antibodies, enzymes, ligands or receptors.^99^ Fifth-generation (5 G) CARs are also based on 2 G CARs with the addition of a cytoplasmic domain derived from cytokine receptors (e.g., IL-2Rβ chain fragment)^154^ or synapse formation proteins (e.g., PDZbm scaffolding anchor domain).^261^ NFAT nuclear factor of activated T cells, IL-12 interleukin 12, IL-2Rβ interleukin 2 receptor beta-chain, JAK Janus kinase, STAT signal transducer and activator of transcription. **c** Numerous approaches to improve the safety and efficacy of CAR T cell therapy. Tandem CARs using bispecific single-chain variable fragments (scFvs) can operate an OR gate and overcome obstacles caused by tumor heterogeneity and antigen loss.^79^ Dual CAR engaging split CARs can perform AND gate to provide and enhance the specificity through targeting multiple antigens.^165^ Switch CARs with ON/OFF switches utilizing small molecule-triggered dimerization or degradation mechanisms can timely control CAR activity and overcome systemic cytokine toxicities of CAR T cells. Switchable CARs are specific to bispecific adaptors, such as folate-FITC, biotinylated antibody, PNE-Fab and Co-LOCKR^96,97,101^ and can direct a universal CAR T cell to target distinct antigens. Split, universal and programmable (SUPRA) CARs consist of a set of leucine-zipper universal CARs (zipCARs) and leucine-zipper scFv (zipFv) domains, which specifically bridge the zipCARs to various antigens. The SUPRA CAR system can fine-tune T cell activation and perform combinatorial logic operations (AND, NOT, OR, AND-NOT).^84,85^ Inhibitory CARs contain inhibitory domains derived from immune checkpoint proteins (PD-1 or CTLA-4), which are able to reduce off-tumor toxicities of CAR T cells by inhibiting T cell activation upon binding an antigen expressed on non-malignant cells.^331^ SynNotch CARs employ a co-expressed synNotch receptor to drive the expression of a CAR to achieve AND logic. The synNotch and CAR can target different antigens, resulting in improved specificity and sensitivity of CAR T cell therapy.^40,190^ deg degron, INH inhibitory domain, TF transcription factor

**Fig. 4:**
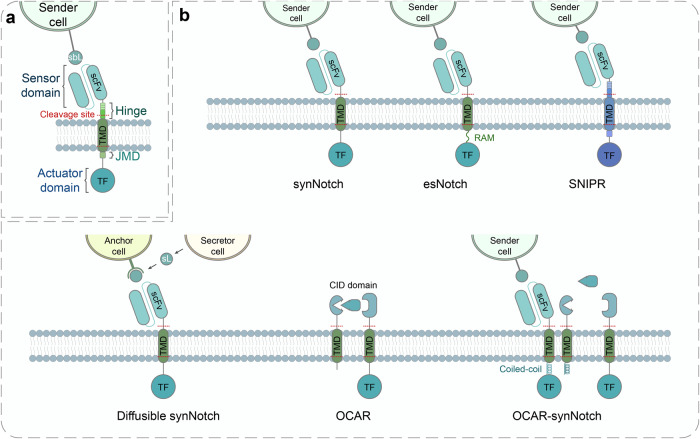
Design and engineering of the synthetic Notch (synNotch) receptor. **a** The architecture of synNotch receptors consists of an extracellular sensor domain, a transmembrane Notch core region and an intracellular actuator domain (transcription factors, TFs). In synNotch receptors, the extracellular and intracellular domains (ICDs) can be completely swapped with diverse recognition domains (scFv, nanobody, or peptide tags) and TFs (transcriptional activators or repressors). The core Notch regulatory region comprises the transmembrane domain (TMD) and multiple proteolytic cleavage sites of wild-type Notch. Ligand binding to synNotch leads to the intracellular proteolytic cleavage and release of the membrane-tethered TF to translocate into the nucleus and regulate gene expression.^31^ sbL surface-bound ligand, scFv single-chain fragment variant, JMD juxtamembrane domain. **b** Evolution of the development of synNotch receptors. (Right, Upper) The modular configuration of prototype synNotch. (Middle, Upper) Enhanced synNotch (esNotch) incorporates an intracellular hydrophobic sequence (QHGQLWF, name as RAM7) derived from native Notch which significantly decreases ligand-independent activation.^192^ (Left, Upper) Synthetic intramembrane proteolysis receptors (SNIPRs) are fully humanized transcriptional receptors through systematic modular engineering of the original synNotch.^42^ (Right, Lower) The diffusible synNotch system can detect diffusible ligands anchored by engineered anchor cells, which enables creating a synthetic morphogen signaling system.^35^ sL soluble ligand. (Middle, Lower) Orthogonal chemically activated cell-surface receptors (OCARs) are engineered by replacing the extracellular sensor domain of synNotch into a chemically induced dimerization (CID) domain, which can achieve small molecule-triggered activation in a *cis* fashion.^193^ (Left, Lower) In OCAR-synNotch system, one part of *cis*-acting OCAR is sequestered by synNotch through the incorporation of coiled-coil dimer-forming peptides into them, which prevents small molecule-induced activation of OCAR when synNotch is in an inactive state. Once synNotch is activated by surface-bound ligands, the sequestered OCAR part is liberated and OCARs can be activated by the addition of inducers, which subsequently enhance synNotch signaling^193^

**Fig. 5:**
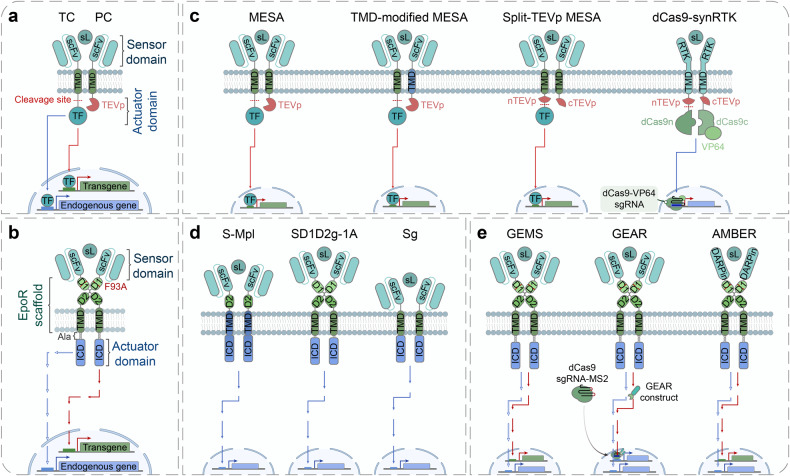
Design and engineering of modular extracellular sensor architecture (MESA) and generalized extracellular molecule sensor (GEMS). **a**, **b** The architecture of MESA (**a**) and GEMS (**b**) comprises the extracellular sensor domain, the transmembrane domain (TMD) and the intracellular actuator domain. The extracellular sensor domain potentially includes single-chain variable fragments (scFvs), nanobodies (Nbs), chemically induced dimerization (CID) proteins, or ligand-binding domains from native receptors. scFv-/Nb-based extracellular sensor domains must bind to non-overlapping epitopes on a single-ligand molecule. **a** The MESA receptor contains two different transmembrane chains, target chain (TC) and protease chain (PC). The TC intracellular domain (ICD) contains an engineered transcription factor (TF) and a protease cleavage sequence between the TMD and the TF. The PC ICD consists of a cognate protease (e.g., tobacco etch virus protease (TEVp) shown here). Ligand binding induces the heterodimerization of the MESA receptor, causing the TEVp to cleave its cognate cleavage sequence on the TC and releasing the TF to translocate into the nucleus and modulate gene expression. Depending on the types of TFs, synthetic promoter-driven transgene can be induced (e.g., tTA) or endogenous gene expression can be regulated (e.g., dCas9-VP64 activator).^47,48^ sL soluble ligand. **b** The GEMS receptor also contains two transmembrane chains, of which the ICDs are derived from various receptor tyrosine kinases (RTKs) and cytokine receptors. Ligand binding induces the dimerization of the GEMS receptor, activating intracellular signaling cascade. By rewiring natural signaling cascades, transgene expression can also be induced.^53^ At the intracellular juxtamembrane region alanine residues are inserted to modulate the conformation of ICD,^201^ thus reducing ligand-independent signaling.^53^ EpoR erythropoietin receptor, D1 EpoR D1 domain, D2 EpoR D2 domain, F93A substitution of phenylalanine at position 93 with alanine, Ala alanine. **c** Evolution of the development of MESA receptors. (Right, Upper) The modular configuration of prototype MESA.^47,48^ (Left, Upper) Systematic evaluation of TMD reveals that the choice of TMD significantly affects MESA performance. The TMD-modified MESA utilizing two different TMDs in the TC and PC can achieve reduced background signals and/or increased ligand-induced signals.^194^ (Right, Lower) In split-TEVp MESA system, computationally optimized split TEVp can be reconstituted via ligand-induced dimerization and therefore restore TEVp function. The split-TEVp enables MESA to achieve low background and high fold induction.^195^ (Left, Lower) dCas9-synRTK (dCas9- and RTK-based chimeric receptor) as an example of dCas9-synRs (synthetic dCas9-based receptors), employs split-dCas9-VP64 and split-TEVp as the intracellular actuator domain, by fusing them to different RTKs. The difference between dCas9-synRTK and split-TEVp MESA is that dCas9-synRTK can only sense native ligands since the extracellular domain and TMD of a dCas9-synRTK are derived from an intact RTK.^196^ nTEVp N-terminal TEVp, cTEVp C-terminal TEVp, dCas9n N-terminal deactivated Cas9, dCas9c C-terminal deactivated Cas9, RTK receptor tyrosine kinase. **d** Engineering of chimeric cytokine receptors to mimic cytokine receptor signaling using scFv and EpoR scaffold. ScFv/c-Mpl (S-Mpl) chimera contains a scFv-based extracellular sensor domain, the extracellular EpoR D2 domain and transmembrane/cytoplasmic domains of cytokine receptors (e.g., c-Mpl).^210–212^ Chimeric cytokine receptor constructs with different combination of the domains containing the extracellular scFv, EpoR scaffold and intracellular domain of cytokine receptors (e.g., gp130). Compared to Sg, SD1D2g-1A additionally contains the extracellular D1D2 domain and one alanine residue at the intracellular juxtamembrane region. But the extracellular D1/D2 domain is dispensable for signaling.^205^ **e** Evolution of the development of GEMS receptors. GEMS should be considered as an evolutionary version of prototype SD1D2g-1A. Through modular engineering, the GEMS platform is able to specifically target a range of soluble ligands and robust transgene expression with high signal-to-noise ratios.^53^ Based on GEMS, generalized engineered activation regulators (GEARs) capitalize on MS2 bacteriophage coat protein (MCP)-nuclear factor fusion proteins and the dCas9/sgRNA-MS2 system to rewire induced receptor signaling to endogenous gene expression.^62^ Advanced modular bispecific extracellular receptors (AMBERs) combine the GEMS system and designed ankyrin repeat proteins (DARPins). The high-throughput binder-screening technology, DARPin, can generate various new binders and endow AMBER with desired sensitivity and specificity towards new inputs.^213^ In addition to customizing target gene expression, GMES and its derivatives inevitably perturb the endogenous gene regulatory network. dCas9, deactivated Cas9; sgRNA, single guide RNA; MS2, MS2 hairpin

In recent years, the construction of evolved synthetic receptors has been facilitated by the rapid development of novel technologies and high-throughput methodologies like directed evolution, rational design and in silico design.^67^ Meanwhile, they are making the implementation of the most classical design-build-test-learn (DBTL) framework in the development process easier than ever. A better combination of the cutting-edge technologies and the DBTL cycle will surely advance a rapid prototyping and optimization of novel synthetic systems for various biomedical applications. Here, we highlight the development and evolution of four of the most advanced single-pass transmembrane synthetic receptors, CARs (Fig. 3a and Table 1), synNotch (Fig. 4a and Table 1), MESA (Fig. 5a and Table 1) and GEMS (Fig. 5b and Table 1).

### Chimeric antigen receptors (CARs)

CARs are a best-known class of synthetic receptor systems, which have already been approved for CAR T cell therapies by the U.S. Food and Drug Administration (FDA)^6,68^ (Supplementary Note 1). They represent a success of advances in synthetic biology to pioneer a new generation of therapeutics, motivating the continuous optimization of CAR-design and the development of new synthetic receptor systems throughout the field.

Currently, CARs have been widely armed in immune cells like T cells, NK cells and macrophages (reviewed in refs. ^5,69,70^). Besides, innate T cells including invariant natural killer T (iNKT) cells, mucosal associated invariant T (MAIT) cells and gamma delta T (γδT) cells have been developed as promising immune effector cells, because they display intrinsic antitumor microenvironment (TME) capacities and minimal risks of graft versus host disease (GvHD) (reviewed in refs. ^71–73^). CAR mainly consists of the extracellular domain (ECD), the transmembrane domain (TMD) and the intracellular domain (ICD) (Fig. 3a). The modular characteristics of its structure confer it advantages to be highly amenable to modification and redesign.

The ECD can be segmented into the signal peptide (SP) and the ligand-binding domain (LBD). The SP prompts the transmembrane receptor protein to be translocated to the plasma membrane, which usually is cleaved from mature CAR protein co-translationally^74,75^ (Supplementary Note 2). The LBD, also called antigen-recognition domain, is typically a single-chain fragment variant (scFv).^76,77^ The scFv is a compact artificial construct that comprises the immunoglobulin light and heavy chain variable regions connected by a flexible linker^78^ (Supplementary Note 2). The scFv is widely adopted in CAR construction due to its compact size, high affinity and specificity for antigen recognition.^77^ By changing the scFv, CARs can specifically recognize different antigens on various cancer cells (e.g., cluster of differentiation 19 (CD19), mesothelin, CEA, B-cell maturation antigen (BCMA), CD38) by one-to-one authentication,^39^ and subsequently activate cancer-killing of CAR T cells. Besides simply replacing the scFv, CARs can be equipped with bispecific antibodies consisting of two different linked antigen-recognition domains (also referred to as ‘tandem CARs’)^79^ (Fig. 3c and Table 1). As a result, tandem CAR T cells can recognize different antigens expressed in a single cancer cell (e.g., human epidermal growth factor receptor 2 (Her2) and interleukin 13 receptor alpha 2 (IL13Rα2),^80^ CD19 and CD20^81^), and therefore reduce the possibility of tumor escape.^82,83^

Recently, Wong and colleagues invented an intriguing generalized CAR platform, the split, universal, and programmable (SUPRA) CAR system for T cell therapy^84^ (Fig. 3c and Table 1). In this system, the conventional CAR architecture is split into two elements: (1) a soluble zipFv by fusing a scFv to a leucine zipper and (2) a universal zipCAR containing the remnant transmembrane and intracellular domains attached to an extracellular cognate leucine zipper.^84^ By adding different zipFv proteins, a unique zipCAR-expressing T cell can retarget different tumor antigens.^84^ Moreover, SUPRA CARs can fine-tune T cell activation and program the Boolean logic operation, which enhances the safety and efficacy of T cell therapy.^84,85^ Alternatively, various switchable CAR systems with bispecific adapters are under way (refs. ^86–95^ and also reviewed in refs. ^96,97^) (Fig. 3c and Table 1). More information about UniCAR is shown in Supplementary Note 3.

As mentioned previously, conventional CARs can only target surface-bound ligands while being unable to sense soluble ligands. To redirect the response of CAR T cells to soluble cues, Chang et al. engineered a CAR that enabled T cells to respond robustly to diverse soluble ligands via dimerization-induced mechanotransduction mechanisms^98^ (Table 1).

The TMD is usually a hydrophobic α-helix that spans the plasma membrane and functions to anchor CAR proteins to the membrane.^68,99^ Besides controlling membrane integration, the TMD also regulates key interactions between CARs, such as assembly, activation and high-order clustering.^68,99,100^ Compared to ECD and ICD, TMD has received relatively less attention and research. Almost all the TMDs used in CARs are derived from natural T cell proteins, such as CD8, CD28, CD4 and CD3ζ.^99,101^ Nonetheless, studies have indicated that the TMDs certainly impact the stability and function of CAR.^102,103^ More recently, Elazar et al., demonstrated that the specific oligomeric state programmed by de novo designed TMDs can tune CAR function and CAR T cell activity, as well as decrease cytokine releasing relative to the commonly used CD28 TMD.^100^

By linking the extracellular antigen-binding and transmembrane domains, the hinge region functions as a linker, providing the flexibility to access sterically hindered epitopes^99,101^ (Supplementary Note 2). Studies have revealed much more crucial roles of the hinge region per se or it being coupled with the TMD than initially expected. These results demonstrate that the length and composition of the hinge can affect CAR functionalities, including fine-tuning CAR signaling activity, improving antitumor efficacy, and lowering cytokine release or neurotoxicity.^102–110^ Therefore, in CAR T cell engineering, it is essential for systematic evaluation and optimization of the hinge and the TMD to ensure optimal performance and reliability.^68,77,99,111–113^

The ICD transmits activation signals upon the antigen’s binding to the ECD. The ICDs of most well-studied CARs contain a CD3ζ-derived signaling moiety, which has three immunoreceptor tyrosine-based activation motifs (ITAMs)^77,114^ (Supplementary Note 4). From the first-generation (1 G) of CAR derived in the 1990s^115^ to now, the structure of CAR per se has been constantly evolving up to the fifth-generation (5 G) (Fig. 3b and Table 1), aiming to increase specificity and minimize off-target toxicity.^114,116^

The first-generation (1 G) CARs only contain ITAMs to provide activating signaling with none co-stimulatory domain^115^ (Fig. 3b and Table 1). Though 1 G CARs were proved to be able to activate T cells and control tumor in mice,^117–119^ they failed to achieve antitumor responses in subsequent clinical studies.^120^ The reason could be the CD3ζ alone is insufficient to activate resting T lymphocytes or trigger the production of optimal amounts of cytokines.^121,122^ To solve these problems, second-generation (2 G) CARs were created by incorporating a costimulatory domain (Supplementary Note 4) on the basis of 1 G CARs (Fig. 3b and Table 1), which enables the activation of a second signal when stimulated by a tumor antigen.^123–128^ This improvement has achieved the enhancement of cytokine production, CAR T cell persistence and antitumor efficacy.^125^

And the third-generation (3 G) CARs are designed to combine multiple costimulatory domains to further enhance CAR T cell potency^129–132^ (Fig. 3b). Although potential benefits including prolonged persistence and increased antitumor efficacy have been demonstrated both in vitro and in vivo,^130–137^ some clinical results did not prove a significant superiority of 3 G CAR T cells to the 2 G CAR T cells.^138–140^ As the available data was obtained from relatively small and heterogenic samples, it is still too early to draw a conclusion, and further large-scale studies are required to fully evaluate their feasibility.

The fourth-generation (4 G) CARs have been engineered from the 2 G CARs to constitutively or inducibly secrete cytokines (e.g., IL-12, IL-7, IL-15, IL-18, and IL-23), enhancing the immune modulating capacities^141–150^ (Fig. 3b and Table 1). Upon CAR-mediated T cell activation, cytokines can be ideally released within targeted tumor locally, alleviating systemic side effects and solving the problem of insufficient production. Since the 4 G CARs can not only improve CAR T cell activation but also hijack host immune cells to enhance tumor killing,^151,152^ the 4 G CAR T cells are also referred to as T cells redirected for universal cytokine killing (TRUCKs).^114,116,151,152^ Compared with conventional CAR T cells, TRUCKs show enhanced expansion and antitumor activity in preclinical studies, especially in animal models of solid tumors. However, in practice, systemic side effects of released cytokines may occur upon entry into circulation. For example, in a clinical study, Rosenberg and colleagues modified tumor-infiltrating lymphocytes (TILs) to express IL-12 under a NFAT-inducible promoter to treat metastatic melanoma. They observed severe toxicity induced in most patients, which is likely attributed to the secreted IL-12.^153^ Here, more data derived from clinical trials are necessary to assess their safety and efficacy.

Distinct from TRUCKs, the immunomodulatory factor expression module of the 5 G CARs is replaced with a novel costimulatory domain which can activate a specific signaling pathway inside equipped CAR T cells^114,116^ (Fig. 3b and Table 1). Based on this excellent principle, several approaches are emerging, of which the addition of IL-2 receptor β-chain (IL-2Rβ) into CARs is a notable example.^154^ Upon activation by the antigen, the extra IL-2Rβ domain allows the activation of the JAK kinase and the STAT3/5 signaling pathways, which can empower CAR T cells to achieve superior antitumor effects with minimal toxicity in mouse models due to a better persistence and expansion in vivo. However, it could potentially increase the risk of CRS, thus requiring to be cautiously addressed in translational studies.^154^

CAR T cell therapy has evolved and gradually matured during the past decades, showing great therapeutic potential in blood and bone marrow cancers. However, challenges remain in CAR-based solid tumor immunotherapy due to tumor heterogeneity, inefficient trafficking and tumor infiltration, and an immunosuppressive TME.^83,155–159^ To facilitate CAR T cell therapy for solid tumors, several strategies have been developed. For instance, dual CAR, tandem CAR and UniCAR systems can recognize more than one antigen, helping mitigate tumor antigen escape. CAR T cells armed with matched chemokine receptor expression can permit trafficking and infiltration, achieving enhanced killing of solid tumors.^160–163^ Also, engineering CAR T cells to express heparanase degrading extracellular matrix (ECM) can promote tumor T cell infiltration and antitumor activity.^164^ In addition, the aforementioned TRUCKs were developed to overcome the drawbacks of the TME on CAR T cell therapy to treat solid tumors by immunomodulatory factors.^142,144^

However, it remains challenging to minimize CAR-immune cells’ off-target and off-tumor toxicity. In this context, the development of next-generation CARs has already been underway^96,99,101^ (Fig. 3c and Table 1). For example, the combinatorial logic control by dual CAR^165,166^ or synNotch CAR^40,167,168^ can enhance tumor targeting specificity via the presence of two or more antigens. On the other hand, a safety control by ON- and OFF-switch CAR can finetune CAR activity^169–172^ and kill-switch CAR can manage the lifespan of CAR T cells.^173,174^ These are promising strategies to improve the safety of CAR T cell immunotherapy in the future.

Furthermore, most CAR T cells under investigation currently are engineered by inserting the CAR construct into autologous T cells without disrupting the endogenous T-cell receptor protein (TCR) gene. Under this condition, the risk of GvHD, which is triggered by human leukocyte antigen (HLA)-TCR mismatching,^175^ can be avoided. To facilitate allogeneic “off-the-shelf” CAR T cell transplantation, T cell receptor α chain (TRAC) deletion using endonucleases, thereby disrupting cell surface expression of the αβ T cell receptor (TCRαβ), can successfully prevent graft-versus-host responses.^175–180^ Recently, CRISPR/Cas-mediated knockin technology enables the precise integration of CAR-encoding gene into *TRAC* locus in human peripheral blood T cells,^181^ which not only facilitates the production of allogeneic CAR T cells,^181–183^ but also enhances T cell potency as the edited T cells outperformed conventionally engineered CAR T cells.^181,184^ However, more recent studies revealed that the endogenous TCR plays a critical role in promoting long-term in vivo persistence of CAR T cells in not only animal models but also patients.^185,186^ These results collectively indicate that it is crucial to balance the intricate effects of removing endogenous TCR from CAR T cells in tumor immunotherapy.

### Synthetic Notch (synNotch) receptors

In-depth studies of Notch receptors provide critical insights into molecular mechanisms of Notch receptors.^187^ And the intrinsic features including modularity and mechanical forces-triggered signaling independent of native ligands make Notch receptors ideally suitable for modular chimeric receptor engineering.^188^ Taking advantage of it, Lim and colleagues created the innovative synNotch receptor system by utilizing the transmembrane core domain of native Notch receptors (Fig. 4a and Table 1), alongside the extracellular sensor domain and intracellular actuator domain.^31,40,41^

Three intriguing works reported the modular synNotch receptors functioning with customized sensing and responsive behaviors in mammalian cells, including T cells.^31,40,41^ Morsut *et al*. demonstrated that synNotch can function orthogonally to control cell differentiation, spatial patterning and Boolean decisions.^31^ Meanwhile, Roybal et al. reported that synNotch can sense tumor antigen and then drive the expression of CARs (synNotch CARs as mentioned above) (Fig. 3c and Table 1), which allows the engineering of AND-gate T cells activated only by dual antigen recognition.^40^ Roybal et al. also reported that synNotch enables CAR T cells to yield customized therapeutic responses like secreting cytokines and antibodies in a very precise and localized manner.^41^ Compared to conventional CARs whose activation drives T cells to produce a native cytokine profile, synNotch can control the expression of extra user-defined cytokines.^41^ A more powerful combination of synNotch receptors with CARs for precise and effective cancer-killing is on the rise, which endows synNotch the great potential of becoming another synthetic receptor system applied in cancer immunotherapy.

Due to its superior performance, the synNotch platform has been widely adopted for designer cell-engineering,^25,33,34^ particularly for the engineering of CAR T cells^40,41,189,190^ and the programming of self-organizing multicellular structures.^31,32,35^ Although there are still challenges and limitations when it comes to practical biomedical applications, researchers are trying hard to figure out possible solutions. First, native Notch receptors have an inherent feature that both *trans*- and *cis*-interaction modes co-exist,^31^ so activation cannot be achieved due to *cis*-inhibition when the cognate ligand presents on the same surface as the receptor does (*cis*).^31,191^ To avoid *cis*-inhibition, the ‘flippase-out’ strategy is employed to achieve the mutually exclusive expression of the synNotch and its ligand in flippase recombinase transgenic *Drosophila*.^191^ Second, synNotch activation requires mechanical forces triggered by surface-bound ligands, making synNotch receptors unable to sense soluble ligands.^31^ To address this, Toda et al. engineered anchor cells which can tether the soluble ligands (e.g., diffusible GFP), thus enabling synNotch receptors to respond to diffusible synthetic morphogens^35^ (Fig. 4b and Table 1). Third, synNotch receptors display a high level of ligand-independent activation,^192^ which is also quite common for other synthetic receptor systems.^20^ Excitingly, a recent work reported an improved version of synNotch, named enhanced synthetic Notch (esNotch) receptor. By adding a native Notch-derived intracellular hydrophobic sequence, an incredible reduction (14.6-fold) in the background activity level was achieved^192^ (Fig. 4b and Table 1). Impressively, Roybal and colleagues have achieved systematic and modular improvements of the synNotch architecture, including modifications of the extracellular sensor domain, TMD, intracellular juxtamembrane domain (JMD) and actuator domain.^42^ The evolved synNotch system is referred as synthetic intramembrane proteolysis receptors (SNIPRs) (Fig. 4b and Table 1), which realized background reductions and enhanced ligand-induced signals.^42^ Meanwhile, SNIPRs can be fully humanized with humanized modules, minimizing the risk of immune rejection.^42^ The use of humanized syn-TFs, including synZiFTRs, not only retains the orthogonality of SNIPRs, but also largely reduces the immunogenic potential for cell-based therapies.^42^

In addition, Fussenegger and colleagues derived an orthogonal chemically activated cell-surface receptor (OCAR) system on the basis of synNotch by substituting conventional protein-specific LBDs with chemically induced dimerization (CID) domains (Fig. 4b). Induced by small molecules, the engineered OCARs on one cell can form heterodimers and trigger signal activation by releasing synTFs.^193^ When the OCAR system is co-expressed with the conventional synNotch on the same cell, one part of OCAR will be sequestered by the synNotch receptor through coiled-coil interactions, making OCAR unable to be activated by the inducer. When synNotch receptor is activated by the presence of sender cells, the OCAR can restore its responsiveness to the inducer and thus further enhance synNotch signaling by adding inducers. Due to their mechanism of action, OCAR systems exhibit an intrinsic off-switch, which might be used as a safety switch in the case of toxicity or malfunction^193^ (Fig. 4b and Table 1).

### Modular extracellular sensor architecture (MESA)

Like synNotch receptors, the modular extracellular sensor architecture (MESA) receptors also take advantage of the proteolytic release of synTFs, which translocate to the nucleus and initiate transcriptional activation to execute orthogonal signals without interrupting endogenous signaling pathways^47,48^ (Fig. 5a and Table 1). But different from synNotch receptors that require mechanical forces induced by surface-tethered ligands,^31^ MESA receptors signal via soluble ligand-triggered receptor heterodimerization to perform the subsequent proteolytic cleavage.^47,48^

Each MESA receptor comprises two different single-pass transmembrane proteins, as their intracellular domains are distinct (Fig. 5a). MESA receptors also comprise three modular domains to transduce extracellular ligand-binding inputs into intracellular transcriptional outputs through synTF-releasing.^47,48^

By engineering the extracellular sensor domain, MESA receptors can be redirected to new ligands.^47,48^ However, the prototype of the MESA architecture also suffers from high level of ligand-independent activation possibly due to transient receptor dimerization during trafficking or on the cell surface.^48^ To address this, Leonard and colleagues have been taking efforts on the improvement of the MESA system, especially to reduce undesired background signals. A systematical strategy was employed to elucidate and refine the MESA architecture, demonstrating that the TMD optimization can lower the background signaling and elevate the target signaling^194^ (Fig. 5c and Table 1). In another study, authors exploited a computational design strategy to use the split-TEVp system for MESA optimization (Fig. 5c and Table 1), achieving both a low background and a high fold induction.^195^ In parallel, a similar dCas9-based synthetic receptor system termed ‘dCas9-synR’ was reported (Fig. 5c and Table 1), which is capable of coupling native input signals with the direct activation of user-defined output response programs.^196^

As discussed above, MESA and dCas9-synR are conceived as cell-surface receptors based on previous studies,^20,28,197^ but a recent study challenged this conclusion.^198^ In this study, the authors showed that both receptor systems did not work with purified protein ligands, but were only activated when using co-transfected ligands or the cell permeable molecule rapamycin as inducers. Therefore, they suspected that these two receptor systems function within the endoplasmic reticulum (ER), and should not be classified into cell-surface receptors.^198^ By carefully checking previous publications, we find in three works relevant to MESA, the authors have shown that MESA receptors could be activated by purified vascular endothelial growth factor (VEGF) protein,^48,62^ and MESA proteins expressed on cell surface were detected by flow cytometry.^48,194^ And yet the inconsistent conclusion drawn by the recent study might attribute to the different components of MESA constructs (e.g., extracellular linker and TMD) used in research,^198^ which could change the expression level of MESA on cell surface as indicated by previous works.^48,194^ Though the early study done by Schwarz et al. showing that MESA could be activated by purified VEGF supported MESA as a cell-surface receptor,^48^ a more recent work from Krawczyk et al. failed to replicate the original data.^62^ Using purified VEGF, few changes (in the range of about 1.1 ~ 1.2 fold) were observed, which strongly indicated that MESA did not achieve cell-surface receptor performance.^62^ Thus, as the bias in the literature exists,^48,62,196,198^ to give a precise characterization of both MESA and dCas9-synR systems, more replication attempts are definitely required.

### Generalized extracellular molecule sensor (GEMS)

A clever insight into the EpoR architecture and signaling mechanisms^199–202^ spurs the development of chimeric cytokine receptors based on EpoR scaffold^203–212^ (Fig. 5d and Table 1). These chimeric cytokine receptors (e.g., SD1D2g-1A) contain extracellular scFv, extracellular EpoR D1D2 as well as TMD domains, and the intracellular domains of cytokine receptors (e.g., glycoprotein 130 (gp130) and EpoR)^205^ (Fig. 5d and Table 1). These chimeric cytokine receptors can employ robust ligand-dependent ON/OFF regulation and mimic the function of the native cytokine receptor system.^205,206^

Based on this prototype, the GEMS system is rationally designed and engineered. GEMS receptors comprise a standard transmembrane scaffold derived from erythropoietin receptor (EpoR) with F93A mutation (Fig. 5b and Table 1), abolishing native ligand (erythropoietin) sensitivity.^53^ Extracellular LBDs can be modularly replaced to sense and respond to a wide range of extracellular soluble ligands. Significantly, the ICDs employ various signaling domains to reroute inputs into distinct endogenous pathways (e.g., JAK/STAT, PI3K/Akt, PLCG and MAPK/ERK).^53^ In recent reports, the GEMS platform has evolved into the superior generalized engineered activation regulators (GEARs)^62^ and advanced modular bispecific extracellular receptors (AMBERs)^213^ (Table 1). The GEAR system combines endogenous signaling-induced synTF nuclear translocation and dCas9-based gene expression via the DNA-binding module (Fig. 5e). Various pathways (e.g., NFAT, nuclear factor kappa B subunit (NFκB), mitogen activated protein kinase (MAPK) or SMAD) activated by diverse receptors including GEMS receptors can be incorporated into engineered GEARs.^62^ The AMBER system is, in short, a type of programmable DARPin- and GEMS-based receptors (Fig. 5e), which inherits the characteristics and advantages of both systems. Thus, AMBERs can be engineered and refined to bind to new soluble ligands in a modular and predictable manner.^213^ For proof of concept, the GEMS and its derivates have been applied to engineering designer cells in vitro and in vivo for disease treatment (Fig. 2d) (refs. ^53,62,213,214^ and reviewed in refs. ^18,19^). Though showing promise in preclinical studies, challenges remain to be addressed before the translation of the GEMS system into clinics. A major challenge is the safety concern relevant to the immunogenicity of designer cells. Recently, Weber and colleagues engineered a material-genetic interface as the safety switch for mammalian therapeutic cells, in which they achieved the control of cell survival by allowing the designer cells to sense whether they were embedded in the hydrogels using a GEMS-based receptor.^215^

As discussed above, synthetic receptors hijacking natural signaling pathways usually inevitably activate endogenous transcription networks to some extent. Using synthetic promotors, GEMS receptors can induce user-defined transgenic target expression.^53^ The advantage is that GEMS receptors can generate high signal-to-noise ratios.^18^ However, the accompanying disadvantage is the perturbation of the gene regulatory network might affect the proliferation and survival of the cells being engineered.

## Synthetic receptor design

The prospect of programming sophisticated customized functions in mammalian cells has fostered the investigations on the design, engineering and iterative improvement of synthetic receptors, which is led by increasing knowledge of the intrinsic structure and molecular acting mechanisms of natural receptors. Currently, primary strategies for synthetic receptor engineering include but are not limited to chimeric/fusion protein, directed evolution, rational design and de novo design (reviewed in ref. ^67^).

Engineering chimeric/fusion proteins means genetically integrating different functional peptides together referring to the architecture of a natural archetype. It is widely used for synthetic receptor engineering. The above-mentioned CARs, synNotch, MESA, GEMS, and chimeric cytokine receptors as well as Tango were originally constructed using this method^67^).

Directed evolution is one of the most popular strategies for protein engineering, which mimics the natural evolution and harnesses the power of mutation and selection.^216,217^ Through iterative cycles of random mutagenesis, followed by selection, engineered DREADDs (designer receptors exclusively activated by designer drugs)^218^ and AMBERs^213^ enable targeting new ligands with high specificity and/or sensitivity (Table 1).

Compared with directed evolution, rational design essentially requires the in-depth understanding of protein structures. Mechanistic insights into receptors have facilitated the rational design and engineering of CARs, synNotch and GEMS receptors. Advanced computation-assisted approaches have aided the rational design of CARs^219,220^ and MESA^195^ as well as other modular sensor-actuator systems^221^ with improved specificity and sensitivity. In silico approaches enable scientists to construct numerous receptor variants and conduct thousands of simulated experiments by computer, which dramatically reduces the amount of candidates to be tested in laboratory experiments.^222,223^

De novo design is an intriguing strategy for protein design with predetermined structures and functions. Distinct from rational design, de novo design aims to generate new proteins from scratch.^224^ With the deepening understanding of the principles of protein biophysics and the development of computational devices and algorithms, scientists have achieved the de novo protein design of synthetic receptors. Currently, various functional proteins, such as protein-binding proteins and TMDs, are de novo designed.^225–231^ And they can be incorporated into synthetic receptor engineering by the modular approach. For example, de novo designed TMDs have been employed in CAR engineering and achieve the outperformance relative to the native CD28 TMD (as discussed above and also in ref. ^100^).

More prominently, Baker and colleagues designed a switchable protein platform from scratch, termed ‘latching orthogonal cage-key proteins’ (LOCKR)^57^ (Table 1). Though not being a cell-surface receptor, LOCKR represents a breakthrough in the de novo design of protein switch, indicating a branch of future direction in synthetic receptor design. In this system, intermolecular cage-key interactions can competitively inhibit intramolecular cage-latch interactions, and thus free the latch peptide from the cage to perform different functions (e.g., binding, degradation, and nuclear export).^57^ Capitalizing on LOCKR technology as the core, engineered modular and tunable protein switches achieve gene expression and even feedback control in cells.^57,58^ The advent of evolutionary technologies opens broad avenues to computationally design, engineer and improve arbitrary synthetic receptor systems.

Different strategies or approaches, including machine learning, could be combined to facilitate the engineering and improvement of synthetic receptors. The latest advances in CAR engineering could be a good example. As afore introduced, intracellular signaling domains of a CAR play vital roles in T cell activation and tumor killing, but only limited sets of signaling domains were explored. To expand the repertoire of CAR signaling domains, a range of studies have been focusing on their systematic optimization. Among them, Goodman et al.^232^ and Si et al.^233^ individually constructed a small library of CARs containing natural or rational recombinant costimulatory domains, and screened out novel costimulatory domains which were proved to achieve enhanced cytotoxicity and T cell persistence, thus improving antitumor efficacy. Meanwhile, high-throughput screening has also been applied to identify new combinations of signaling domains^234^ or new synthetic signaling domains via shuffling or recombination.^235,236^ Of note, Lim and colleagues incorporated machine learning in their study, and took advantage of trained neural networks to predict the cytotoxicity and stemness of CARs with synthetic signaling domains.^236^

**Fig. 6:**
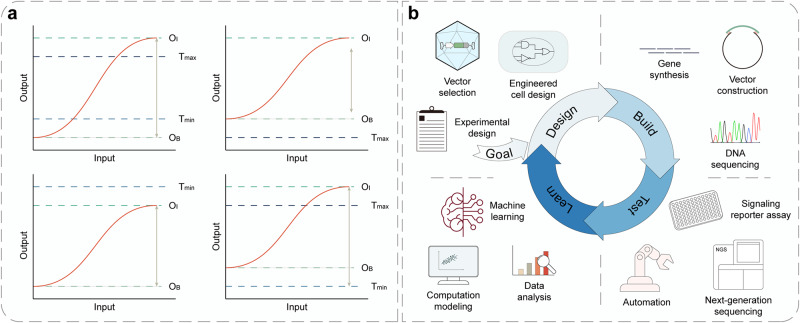
Metrics- and “design-build-test-learn” (DBTL) cycle-based framework for synthetic receptor engineering. **a** Performance metrics can be used for quantitative assessment of the performance of synthetic receptor systems. Signaling output can be quantified by reporter fluorescence measurement, luciferase assay or various other approaches. The performance of a synthetic receptor is determined not only by its background signal and signal-to-noise ratio, but also by the therapeutic thresholds in practical applications. An optimal receptor should exhibit a low background signal (O_B_) which is lower than the minimum therapeutic threshold (T_min_), and meanwhile exhibit a high induced output signal (O_i_) (Right, Upper). A synthetic receptor exhibiting low O_B_ and high O_i_ may still fail in practical applications, which might be due to O_B_ > T_max_ (maximum therapeutic threshold) (Right, Lower) or O_i_ < T_min_ (Right, Lower). In another case when O_B_ > T_min_ (Left, Lower), leaky expression should be cautious of. **b** A modified DBTL-based framework can direct researchers to choose or engineer synthetic receptor systems for their application in cell therapy or gene therapy. A “goal” step is to define design objectives for engineered cell or gene therapy and the standards to quantify the performance of synthetic receptors using performance metrics. In the DBTL cycle, apart from conventional approaches, more advanced and powerful approaches like computation-guided design, high-throughput automation techniques, machine learning and computational modeling can further accelerate the engineering and improvement of synthetic receptors for better clinical applications. The figure is adapted from refs. ^20,239^

Increasing approaches are boosting the development and improvement of various synthetic receptors (Table 1). For practical use, selecting and optimizing synthetic receptors for a specific application could be greatly facilitated by the generalized and systematic framework presented in Fig. 6 (also reviewed in detail in ref. ^20^). Defining the goal is the first and crucial step before entering into the DBTL cycle. It includes narrowing down options of synthetic receptors adapting to engineered cell or gene therapy by specifying the required characteristics and setting performance criteria for the expected synthetic receptors. Notably, performance metrics can be adopted to quantitively evaluate the performance of synthetic receptors in different application scenarios^20^ (Fig. 6a).

Driven by defined design objectives, researchers could follow the DBTL pipeline to design and engineer synthetic prototypes, test and optimize the performance in a befitting model (e.g., cell lines), and further validate the performance in a practical context (e.g., engineered cell therapy or gene therapy in animal models) (Fig. 6b). Of note, in the “design” step, choosing different module candidates is of vital importance, which could significantly impact the properties of synthetic receptors. For example, to force the expression of synthetic receptors on cell surface, both SP and TMD are essential (discussed above), which also influence the cell-surface expression levels of receptors. Moreover, data-driven computation-guided design can be incorporated to achieve a more efficient rational design of synthetic receptors. The “build” and “test” steps are traditionally laborious, involving series of construct engineering and assessment of performance metrics. The high-throughput automation techniques developing rapidly can not only increase productivity but also reduce human errors in this process.^237,238^ In the “learn” step, researchers can decipher the data generated and create blueprints of synthetic receptors based on it. Usually trial-and-error approaches are employed to determine the optimum design subsequently. Currently, advanced computational modeling and machine learning are starting to play a central role to process and learn from masses of biological data, achieving a new paradigm of predictive design. By bridging the gap between the “learn” and “design” stages, they can greatly accelerate the DBTL cycle.^239^

## Conclusions and perspective

The design and engineering of synthetic receptors have achieved great advancements, and the constant evolution of synthetic receptor systems accelerates their biomedical applications and clinical translations. As the most successful case, CARs are at the forefront, with six CAR T cell therapies already approved by the FDA^6,68^ and hundreds more undergoing clinical studies (ClinicalTrials.gov). In addition, other CAR-engineered cells including CAR NK and CAR macrophage cells are also being widely investigated in translational researches.^5,240–242^

Looking forward, an important goal of the field is to uncover the principles of design to guide the rational design of synthetic receptors with desired properties and functions. A growing number of reports have taken advantage of computational designing strategies for engineering and improving synthetic receptor systems like CARs,^219,220^ MESA^195^ and de novo protein switches.^57,58^ Among them, de novo protein design is further ahead, as different kinds of non-natural functional proteins have been created from scratch.^225–231^ Knowledge-driven and data-driven computational approaches have established interpretable models for de novo protein design.^243–249^ As discussed above, de novo designed proteins have already been incorporated into synthetic receptor engineering.^57,58,100^ We anticipate that various valuable synthetic receptors can be designed by de novo design strategies^224,250^ or by generative language models in future.^251^

**Fig. 7:**
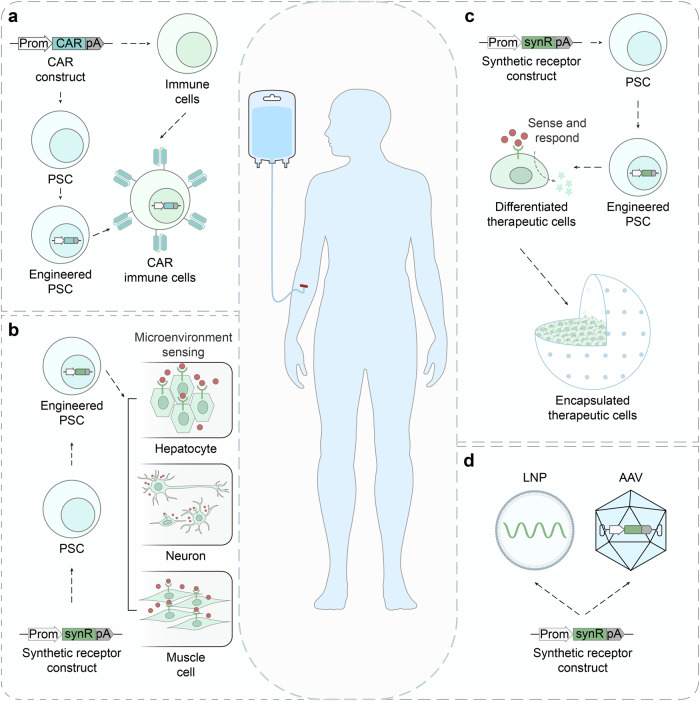
The expanding potential therapeutic applications of synthetic receptors. **a** A variety of immune cells, such as T cells, innate T cells, NK cells, macrophages, dendritic cells and myeloid cells, can be directly isolated from patients and genetically engineered with CARs to enhance their antitumor capacity.^68,332^ Alternatively, these immune cells can be differentiated from CAR-engineered pluripotent stem cells (PSCs) as ‘off-the-shelf’ products. After being infused back into patients, these engineered immune cells interact with antigens on the tumor cells, leading to the activation of CAR immune cells to achieve cancer-killing.^70,333^ **b**, **c** Synthetic receptors could be integrated into PSCs to enhance original or program novel functionalities of the differentiated derivates for developing next-generation cellular therapeutics. **b** PSCs can differentiate into various cell types, like hepatocytes, neurons, muscle cells, etc., which are suitable for transplantation. One could imagine after being transplanted, cells engineered with synthetic receptors could sense and respond to host microenvironmental cues to promote the survival, proliferation and enhance tissue repair or regeneration.^30^ **c** Differentiated derivates from synthetic receptor-engineered PSCs might also be encapsulated and implanted in patients to avoid immunogenicity. These implantable therapeutic cells can sense various serum biomarkers (e.g., glucose, uric acid or thyroid hormone) and then trigger the activation of the corresponding therapeutic functions.^16^ **d** Synthetic receptor systems can be further designed and engineered with compact size and lower immunogenicity easier for in vivo gene therapy.^42,64^ These suitable synthetic receptor constructs can be delivered by non-viral vectors (e.g., lipid nanoparticle (LNP))^334,335^ or viral vectors (e.g., AAV)^27^

Importantly, selecting functional synthetic receptor systems and integrating them with ‘chassis cells’ can potentially push the boundaries of synthetic receptor applications and develop novel cell therapeutics (Fig. 7). For example, the application of CARs in engineered-T cells has enhanced the specific tumor killing ability^68^ (Fig. 7a). More importantly, a combination of different synthetic receptor systems can exhibit synergistic effects and further enhance their performance, as studies have shown that synNotch CAR T cells exhibit a significantly enhanced safety and antitumor efficacy by combinatorial antigen recognition^40,167,168,189,252^ or ultrasensitive antigen-density sensing.^190^ As engineered stem cell therapy emerges, synthetic receptors can also be applied to engineer pluripotent stem cell-derived cell products^3,70,175^ (Fig. 7b, c), potentially being used to enhance their survival and engraftment ability via programmed communications with the host microenvironment^2,3,253,254^ (Fig. 7b). However, since some precautionary data and critical attitudes about stem cell therapy exist, future trials are needed to further investigate the safety and efficacy of stem cell therapy per se.^2,255^ Meanwhile, concerns over the safety of both genetic materials (e.g., transgene immunogenicity) and genetic modifications introduced also require to be carefully addressed in preclinical and clinical studies.^3^

Moreover, synthetic receptors can also program in vivo gene therapy with an improved safety and efficacy (Fig. 7d). One promising approach for in vivo gene therapy is to employ adeno-associated virus (AAV) systems to deliver therapeutic genetic materials into the human body.^256,257^ Although AAVs have been increasingly used in clinical trials as novel gene therapies,^258^ their limited packaging capacity (~4.7 kb) hinders the loading of most synthetic receptors described above. To meet the clinical demands, designing or refining synthetic receptors with a compact size is required, and already being investigated.^42,64^ In addition, humanizing synthetic receptors with minimal immunogenicity is also of vital importance in advancing gene therapy as well as engineered cell therapy.^42,64^

In conclusion, modular synthetic receptors with desired functions have been engineered and applied to therapeutic applications. Meanwhile, advances keep facilitating and accelerating the development and evolution of synthetic receptor systems, making the field to shift from the prior trial-and-error mode to more knowledge- and data-driven modes. Briefly, we expect that rapid progress in synthetic receptor biology will keep revealing exceptional new openings to program gene therapies and engineered-cell therapies that have been unreachable by established approaches.

### Supplementary information

Supplementary Material
