# Supplementary material for: Programmable synthetic receptors: the next-generation of cell and gene therapies
Source: Signal Transduct Target Ther. 2024 Jan 3;9:7. doi: 10.1038/s41392-023-01680-5 (PMC10761793; doi:10.1038/s41392-023-01680-5)
Supplement: Supplementary file 1 — Supplementary Material [file 41392_2023_1680_MOESM1_ESM.docx]

**Supplementary Information for**

**Programmable Synthetic Receptors: the next-generation of cell and gene therapies**

**Author list**

Fei Teng,^1,*,#^, Tongtong Cui,^2,3.*^, Li Zhou,^1,2,3^, Qingqin Gao,^1,2,3^, Qi Zhou,^1,2,3,4,#^, Wei Li,^1,2,3,4,#^

**Affiliations**

^1^ University of Chinese Academy of Sciences, Beijing 100049, China

^2^ State Key Laboratory of Stem Cell and Regenerative Biology, Institute of Zoology, Chinese Academy of Sciences, Beijing 100101, China

^3^ Institute for Stem Cell and Regeneration, Chinese Academy of Sciences, Beijing 100101, China

^4^ Beijing Institute for Stem Cell and Regenerative Medicine, Beijing 100101, China

^*^ These authors contributed equally

^#^ Correspondence: [liwei@ioz.ac.cn](mailto:liwei@ioz.ac.cn) (W.L.), [zhouqi@ioz.ac.cn](mailto:zhouqi@ioz.ac.cn) (Q.Z.) and [tengfei@ucas.ac.cn](mailto:tengfei@ucas.ac.cn) (F.T.)

**This file includes:**

Supplementary Note 1

Supplementary Note 2

Supplementary Note 3

Supplementary Note 4

Supplementary Table 1

Supplementary Table 2

Supplementary Table 3

**Supplementary Note 1. FDA-approved CAR T cell therapies**

Chimeric antigen receptor (CAR) T cell therapy is an advanced type of immunotherapy of cancers. Autologous or allogeneic T cells can be engineered with CARs to specifically recognize and eliminate target cancer cells. Since 2017, six CAR T cell therapies have been approved by the U.S. Food and Drug Administration (FDA)^1,2^. All of them are autologous and are approved for treatment of blood cancers, including lymphomas, leukemia and myeloma^1,2^.

As shown in the Supplementary Table 1, the six CARs belong to the so-called second generation (2G) CARs, which contain one co-stimulatory domain (CD28 or 4-1BB) and a signaling domain from CD3ζ. In addition, all registered treatments and most clinical trials are based on 2G CARs. There are several reasons that might explain this trend. First, it has been clearly demonstrated that 2G CARs outperform 1G CARs in preclinical and clinical studies, largely attributing to improved long-term persistence and enhanced antitumor efficacy^3^. Second, from the emergence of 2G CARs to their successful approval, it took about two decades^2^. During the past two decades, extensive preclinical and clinical studies have been performed to support the safety and efficacy of CAR T cell therapy. Therefore, based on 2G CAR prototype, it is relatively easier to carry out new clinical research and get investigational new drug (IND) application approved. Third, 3G CAR T cells did not achieve significantly enhanced efficacy compared to 2G CAR T cells in clinical reports^4-6^, though 3G CAR T cells exhibited increased persistence and better proliferation^7-9^. Fourth, the favorable properties of these relatively newly invented CAR constructs, including 3G, 4G, 5G and the next-generation CARs, make them a promising alternative to 2G CARs, but studies are still required to further address their safety and efficacy. Fifth, the inclusion of additional signaling domains or constructs might bring new hazards, resulting in CAR T cell depletion, transformation into malignant clones, or toxicities associated with induced cytokines. These concerns should be addressed in ongoing and future clinical trials when new-generation CARs are implicated.

**Supplementary Table 1. FDA-approved CAR T products.**

| **Generic Name** | **Brand Name** | **Approval Date** | **Targeted Antigen** | **Receptor Type**  **(scFv-Hinge/TMD-CD-SD)** | **Target Patients** | **References** |
| --- | --- | --- | --- | --- | --- | --- |
| Tisagenlecleucel | Kymriah™ | 2017.08 | CD19 | FMC63-CD8α_H/T_-4-1BB-CD3ζ | Pediatric and young adult patients (aged up to 25 years) with relapsed and/or refractory B‑cell precursor acute lymphoblastic leukemia | ^10-16^ |
| Axicabtagene ciloleucel | Yescarta™ | 2017.10 | CD19 | FMC63-CD28_H/T_-CD28-CD3ζ | Adult patients with relapsed or refractory diffuse large B-cell lymphoma | ^15,17,18^ |
| Brexucabtagene autoleucel | Tecartus™ | 2020.07 | CD19 | FMC63-CD28_H/T_-CD28-CD3ζ | Autologous T cell therapy for relapsed or refractory mantle cell lymphoma | ^19,20^ |
| Lisocabtagene maraleucel | Breyanzi® | 2021.02 | CD19 | FMC63-IgG4_H_-CD28_T_-4-1BB- CD3ζ | Adult patients with relapsed or refractory large B cell lymphoma | ^21,22^ |
| Idecabtagene vicleucel | Abecma® | 2021.03 | BCMA | C11D5.3-CD8α_H/T_-4-1BB-CD3ζ | Adult patients with relapsed or refractory multiple myeloma | ^23-26^ |
| Ciltacabtagene autoleucel | Carvykti™ | 2022.02 | BCMA | 2xVHH-CD8α_H/T_-4-1BB-CD3ζ | Adults with relapsed or refractory multiple myeloma | ^27-29^ |

Abbreviation: CD19, cluster of differentiation 19; BCMA, B-cell maturation antigen; scFv, single-chain fragment variable; VHH, variable heavy domain of heavy-chain antibody, also known as nanobody; H (subscript), Hinge domain; TMD or T (subscript), transmembrane domain, CD, co-stimulatory domain; ITAM, immunoreceptor tyrosine-based activation motif; CD8α, CD8 subunit alpha; 4-1BB, also known as CD137 or tumor necrosis factor receptor superfamily 9 (TNFRSF9); IgG4, immunoglobulin G4; CD3ζ, CD3 zeta chain, also known as CD247.

**Supplementary Note 2. Signal peptide and flexible linker for CAR**

Chimeric antigen receptor (CAR) protein is a type I transmembrane protein that must be anchored in the plasma membrane to exert its function by recognizing the target antigen ^1^. Therefore, the extracellular domain of a CAR precursor protein comprises three components: a signal peptide (SP), an antigen-recognizing domain and a spacer^1,30^.

As a type I transmembrane protein, the N-terminal SP directs the nascent CAR protein across the endoplasmic reticulum (ER) membrane and is then cleaved off by an enzyme called “signal peptidase”, while the downstream transmembrane domain (TMD) serves as a membrane anchor to insert the CAR protein into the ER membrane^31-33^. Subsequently, CAR proteins in the ER membrane will traffic to the plasma membrane and function as a transmembrane receptor^34^. Hence, the SP might affect CAR protein expression and function on the plasma membrane, though we have not found comprehensive assessment of the SP optimization.

Here, we summarize SP sequences currently used in CAR engineering (Supplementary Table 2), and show that these SPs are derived from transmembrane proteins (e.g., CD8, CSF1R, GM-CSFR, and INFγR1) and secretory proteins (GM-CSF, immunoglobulin and IL-2). In a recent study, the effects of four SPs from human CD8α, GM-CSFRα, IL-2 and mouse Igκ were assessed on CAR expression and antitumor activity using an *in* *vitro* model^35^. The authors revealed that IL-2 SP outperform three other SPs in promoting CAR expression on T cell plasma membrane, cytokine expression level and CD19^+^ cell killing ability^35^. In another study, six SPs were used to engineer secretory PD-1-neutralizing scFv in CAR T cells, and results indicated that SPs could affect the secreting capacity of anti-PD-1 scFv^36^. Though not being direct evidence, it suggests that the choice of SP might influence the expression level of CARs on the plasma membrane, as type I transmembrane proteins and secretory proteins adopt common co-translation pathways and secretory pathway^33,34^.

**Supplementary Table 2. Signal peptide sequences used in CAR constructs.**

| **Signal peptide** | **Species** | **Sequence** | **References** |
| --- | --- | --- | --- |
| Cluster of differentiation 8 subunit alpha (CD8α) | Human | MALPVTALLLPLALLLHAARP | ^7,9,10,37-61^ |
| Colony-stimulating factor-1 receptor (CSF1R) | Human | MGPGVLLLLLVATAWHGQG | ^62^ |
| Granulocyte-macrophage colony-stimulating factor receptor alpha chain (GM-CSFRα)^*^ | Human | MLLLVTSLLLCELPHPAFLLIP | ^63-69^ |
| GM-CSF^#^ | Human | MWLQGLLLLGTVACSIS | ^70,71^ |
| Immunoglobulin heavy-chain | Human | MEFGLSWLFLVAILKGVQC | ^72-75^ |
| Interleukin-2 (IL-2) | Human | MYRMQLLSCIALSLALVTNS | ^76^ |
| CD8α | Mouse | ASPLTRFLSLNLLLLGESIILGSGEA | ^48,50,77^ |
| Immunoglobulin kappa light chain (Igκ) | Mouse | METDTLLLWVLLLWVPGSTGD | ^78-81^ |
| Interferon gamma receptor 1 (INFγR1) | Mouse | MGPQAAAGRMILLVVLMLSAKVGSG | ^82^ |

^*^ Also known as colony stimulating factor 2 receptor subunit alpha (CSF2Rα)

^#^ Also known as CSF2

There are two flexible linkers existing in the extracellular domain. The spacer linking the antigen-recognizing domain and the transmembrane domain (TMD), also known as hinge domain (HD)^83,84^, is usually overlooked when evaluating CAR functionality. But evidence has indicated its important role in CAR engineering. The HD serves as a flexible linker that provides the flexibility to access antigens on the surface of target cells. Currently, the most frequently used hinge domains are derived from immunoglobulin molecules (e.g., IgG1 and IgG4), or proteins naturally expressed on T cells (e.g., CD8 or CD28)^83,84^. Here, we summarize comparative studies to illustrate HD selection for CAR optimization (Supplementary Table 3).

**Supplementary Table 3. Comparative overview of hinge domains used in CAR constructs.**

| **Hinge domain** | **Activation signal** | **Conclusions** | **References** |
| --- | --- | --- | --- |
| IgG1_Hinge-CH2-CH3_ vs. IgG1_Hinge-CH2∆-CH3_ | CD28-CD3ζ | - IgG1_Hinge-CH2-CH3_ mediates unintended binding of CAR T cells to IgG Fc receptors (FcγRs) on innate immune cells, resulting in off-target activation of CAR T cells - FcγR binding activates innate immune cells to secret inflammatory cytokines, resulting in unintended innate immune response - Modifications of IgG1 CH2 residues abolish cross-activation of CAR T cells and innate immune cells | ^85^ |
| IgG4_Hinge-CH2-CH3_ *vs.* IgG4_Hinge-CH3_ *vs.* IgG4_Hinge_ | CD28-CD3ζ | - HD length do not affect the CAR expression level - Cytokine production and proliferation of CAR T cells: short spacer > intermediate spacer > long spacer - Antitumor efficacy: short spacer > intermediate spacer > long spacer | ^86^ |
| IgG4_Hinge-CH2-CH3_ *vs.* IgG4_Hinge-CH3_ *vs.* IgG4_Hinge_ | 4-1BB-CD3ζ | - CARs with IgG4_Hinge-CH2-CH3_ are functional *in* *vitro* but lack antitumor activity *in* *vivo* - CH2 region can interact with FcγRs on the innate immune cells, leading to activation-induced cell death *in* *vivo* - CAR T cells with CH2 region removing can improve cell proliferation and persistence, as well as antitumor effects *in* *vivo* - Introducing mutations into long spacer can also improve CAR T cells function *in* *vivo* | ^87^ |
| IgG4_Hinge-CH2-CH3_ *vs.* IgG4_Hinge-CH2(EQ)-CH3_ ^*^ *vs.* IgG4_Hinge-CH3_ | CD28-CD3ζ |  | ^64^ |
| IgG4_CH3_ *vs.* IgD vs. none | CD28-CD3ζ | - HD incorporation (both IgG4 and IgD) can enhance CAR T cell proliferation *in* *vitro* - HD can enhance antitumor activity of specific CARs | ^88^ |
| CD8α_H/T_ *vs.* CD28_H/T_ | CD28-CD3ζ | - CAR T cells with CD8α_H/T_ or CD28_H/T_ exhibit similar antitumor capacity in mice - CD8α_H/T_ expressing CAR T cells produce lower levels of cytokines - CD8α_H/T_ expressing CAR T cells undergo less activation induced cell death - CD28_H/T_-bearing CARs have stronger activation stimulus - CD28 HD is more likely to promote the dimerization of CARs | ^89^ |
| CD8α_H/T(71AA)_ *vs.* CD8α_H/T(75AA)_ *vs.* CD8α_H/T(82AA)_ *vs.* CD8α_H/T(86AA)_ *vs.* CD8α_H/T(96AA)_ | 4-1BB-CD3ζ | - CAR T cells with CD8α_H/T(86AA)_ have lower cytokine production but higher proliferation and persistence ability than CAR T cells with CD8α_H/T(71AA)_ and CD8α_H/T(75AA)_ - CD8α_H/T(86AA)_ expressing CAR T cells are effective in treatment of refractory B cell lymphoma in patients - CD8α_H/T(86AA)_ expressing CAR T cells do not cause neurological toxicity or severe cytokine-release storm (CRS) | ^90^ |
| CD8α_Hinge_ *vs.* IgG4_Hinge_ *vs.* IgG4_Hinge-CH3_ *vs.* IgG4_Hinge-CH2-CH3_ | 4-1BB-CD3ζ | - CAR T cells with IgG4_Hinge_ outperform CAR T cells with other types of spacers in targeting low antigen expressing cancer xenografts in mice - CAR T cells with IgG4_Hinge_ with CD28_TMD_ are more polyfunctional (co-secretion of 2 or more cytokine/chemokines), contributing to the long-term immune response - IgG4_Hinge_- and CD28_TMD_-bearing CARs attend to dimerize to enhance CAR T cell signaling and function | ^91^ |

Besides, another neglected flexible linker between the heavy and light chain can also affect the conformation of the scFv, thereby changing the functional properties of CARs. The commonly used linkers in scFvs are repeats of glycine and serine residues, -(GGGGS)n-, helping provide the flexibility necessary for antigen recognition and maintain high stability in aqueous solutions^92^. It has been previously shown that a long flexible linker was able to increase the activity of scFv toward targeting antigen^93^ while a short one favored multimerization of scFv^94^.

Though the scFv linker has not been implicated in affecting CAR T cell efficacy in preclinical studies^95,96^, a recent study demonstrated that the linker length could significantly determine the clinical outcomes of CAR T cell therapy through detailed interrogation^97^. After analyzing the composite outcomes of two pilot clinical trials (NCT02588456 and NCT02650414) applying anti-CD22 CAR T cell therapy in patients with acute lymphoblastic leukemia (ALL)^98,99^, researchers found that unexpectedly lower response rates were attributed to the length of scFv linker^97^. More importantly, the authors performed comparative studies using two anti-CD22 CARs with short (GGGGS, CAR22-short) or long ((GGGGS)_4_, CAR22-long) scFv linkers, and revealed that the short scFv linker promoted CAR22 multimerization, resulting in antigen-independent T cell activation and enhanced T cell function^97^. Interestingly, the short scFv linker triggered tonic signaling, which was beneficial for 4-1BB-costimulated CD22 CAR T cells, as replacing 4-1BB with CD28 led to T cell exhaustion and dysfunction^97^. Moreover, the beneficial effect of short scFv linker is not a universal phenomenon, because CD19 CAR T cells with the short scFv linker did not induce observable CAR clustering and corresponding tonic signaling^97^.

**Supplementary Note 3. Universal CAR (UniCAR).**

Traditional CARs have a fixed design, making CAR T cells can only target one antigen. Even tandem CARs, capable of targeting two or three epitopes, also hold a fixed design. Therefore, to target a new antigen even on the same cancer cell, a new CAR must be re-engineered. Besides, due to this rigid design, the magnitude of cell activation and cytokine release after CAR T cell infusion could be unmanageable, which might trigger CRS and associated neurotoxicity^100,101^. In order to increase the versatility of CAR T cells, split universal CAR (referred as UniCAR) configurations were designed, in which the antigen recognition domain is split from the signaling domain of the conventional CAR. Hence, the target antigen can be redirected readily with no requirement of re-engineering the CAR T cells.

In a UniCAR system, the conventional CAR architecture is split into two components: (1) a universal receptor containing the remnant transmembrane and intracellular domains with an extracellular binding moiety and (2) a soluble bispecific adaptor that binds to both the target antigen and the cognate binding moiety of the universal receptor^102^. Currently, several UniCAR systems have been engineered using different dimerizing platforms including leucine zippers^103,104^, biotin-avidin systems^105-107^, and neo-epitope tagging systems including fluorescein isothiocyanate (FITC)^108-111^, peptide neo-epitope (PNE)^44,49^, and benzylguanine^112^.

UniCARs are highly programmable, which enables them to regulate the signal strength, expand tumor-targeting repertoire, and thereby increase the safety and efficacy of CAR T cell therapy^102^. Nowadays, various UniCAR designs are emerging, including switchable CAR systems (refs.^44,49,105,108-111,113-115^ and also reviewed in refs.^102,116,117^) (Fig. 3c and Table 1) and the most versatile split, universal, and programmable (SUPRA) CAR system^103^ (Fig. 3c and Table 1). For example, in the SUPRA CAR system, a variety of orthogonal sets of leucine zipper universal CARs (zipCARs) and leucine zipper fused scFvs (zipFvs) make it highly programmable. By simply adding a zipFv, the cognate zipCAR T cells can be activated. By changing zipFvs with different antigen-specific scFvs, the same zipCAR T cells can retarget different tumor antigens and thereby counteracting antigen escape without re-engineering CAR T cells. Moreover, the activated CAR T cells can be shut down by ceasing the administration of zipFv or by adding a competitive zipFv without antigen-specific scFv. By adjusting the concentration of zipFv or the affinity between leucine zipper pairs, the strength of CAR T cell activation can be modulated to mitigate toxicity. More significantly, SUPRA CAR systems can be flexibly designed for combinatorial logic operations (OR, AND, NOT) against multiple antigens to combat tumor relapse^103,104^.

Although UniCAR systems are versatile and programmable, the increased complexity brings challenges for their optimization and may also reduce the efficacy in clinical application. First, UniCAR T cell therapy relies on adding exogenous adaptors, which might cause potential immunotoxicity^102,117,118^. Second, the *in* *vivo* application of UniCAR T cells requires repeated administration of high-dose of adaptors, which also increases the difficulty of practical implementation^102,117,118^. Therefore, clinical applications of the bispecific adaptors *per* *se* should involve additional clinical trials and regulatory examinations for safety and efficacy. In addition, the combination of UniCAR designs into allogeneic “off-the shelf” T cells (as discussed in the main text and also refs.^102,119^) would further broaden the clinical application of universal CAR T cells, possibly able to target any tumor types and meanwhile lower the cost of CAR T cell therapy.

**Supplementary Note 4. The intracellular signaling domain of CAR.**

**CD3ζ and FcεRIγ**

Early studies used CARs containing the ITAMs derived both from the CD3ζ-chain and the FcεRIγ-chain as the activating domain (as reviewed in ref.^120^). However, the outperformance of the CD3ζ-chain over the FcεRIγ-chain makes CD3ζ-based CARs more prevalent in CAR T cell engineering in current studies^30,121^. Haynes *et al.* showed that CD3ζ-based CARs prompted superior cytokine release and toxicity *in* *vitro*, as well as high antitumor efficacy in mice^122^. Then, Heuser *et al*. demonstrated that CAR T activation mediated by CD3ζ-chain was more efficient than by FcεRIγ-chain upon prolonged propagation *in* *vitro*, and FcεRIγ-based CARs exhibited a higher antigen density threshold for CAR reactivity^123^. Recent studies through either truncation or mutation to decrease the number of active ITAMs demonstrated that decreasing the number of functional ITAMs reduced the signal strength and function of CAR T cells, but resulted in increased persistence and decreased T cell exhaustion^47,124,125^. Sadelain and colleagues also found that CAR T cells expressing only one (the most membrane-proximal) CDζ ITAM were able to function equivalently to their wild-type counterparts in killing tumors with high CD19 antigen density^47^, which was further demonstrated in Majzner’s work^126^. Moreover, Majzner *et al.* also revealed that CAR T cells with more CDζ ITAMs achieved greater antitumor activity by doubling the CDζ domain, and the enhanced signaling strength in CAR T cells enabled recognition of low-antigen-density cells *in* *vivo*, which was consistent with the results generated from *in* *vitro* models^127^. Intriguingly, Wu *et al.* identified an inhibitory ITAM derived from CD3ε and demonstrated that incorporation of this inhibitory CD3ε into CD3ζ-based CARs led to reduced cytokine production but enhanced T cell persistence, resulting in an improved antitumor activity^67^. These results collectively indicate the importance of ITAM in CAR design to enhance therapeutic profiles.

**CD28 and 4-1BB**

The 2G CARs contain one costimulatory domain, with CD28 and 4-1BB (CD137) best-known^128^. The addition of the CD28^37,129-131^ or 4-1BB^39^ costimulatory domain achieved the enhanced T cell proliferation and increased IL-2 secretion. And subsequent studies further demonstrated the superior antitumor activity of 2G CARs to 1G CARs in mice^73,132,133^ and patients^3^. Preclinical and clinical studies revealed that CARs with a CD28 or 4-1BB costimulatory domain confer T cells with similar antitumor activity. Meanwhile, it was unveiled that CD28 and 4-1BB differ in expansion pattern and intracellular signaling. Generally, CD28-costimulated CARs are more likely to elicit a faster signaling and higher levels of cytokine production, which may trigger higher risks of clinical toxicities. 4-1BB-costimulated CARs promote a more progressive response and greater persistence of T cells that may compensate the less immediate potency, which confers a more favorable safety profile^134-136^. Since neither costimulatory domain is consistently superior for antitumor effects in all aspects, more comprehensive comparisons are necessary, particularly in clinical trials. Meanwhile, several other costimulatory domains have already been evaluated, including those derived from inducible T cell co-stimulator (ICOS)^137-139^, OX-40^137,140^, CD27^141,142^, MyD88/CD40^143^, and natural killer group 2 member D (NKG2D)^144-146^. Their performances in CAR function has been reviewed elsewhere^83,147^.

**References**

1 Mazinani, M. & Rahbarizadeh, F. CAR-T cell potency: from structural elements to vector backbone components. *Biomark. Res.* **10**, 70, (2022).

2 Labanieh, L. & Mackall, C. L. CAR immune cells: design principles, resistance and the next generation. *Nature* **614**, 635-648, (2023).

3 Savoldo, B. *et al.* CD28 costimulation improves expansion and persistence of chimeric antigen receptor-modified T cells in lymphoma patients. *J. Clin. Invest.* **121**, 1822-1826, (2011).

4 Morgan, R. A. *et al.* Case report of a serious adverse event following the administration of T cells transduced with a chimeric antigen receptor recognizing ERBB2. *Mol. Ther.* **18**, 843-851, (2010).

5 Till, B. G. *et al.* CD20-specific adoptive immunotherapy for lymphoma using a chimeric antigen receptor with both CD28 and 4-1BB domains: pilot clinical trial results. *Blood* **119**, 3940-3950, (2012).

6 Enblad, G. *et al.* A phase I/IIa trial using CD19-targeted third-generation CAR T cells for lymphoma and leukemia. *Clin. Cancer Res.* **24**, 6185-6194, (2018).

7 Carpenito, C. *et al.* Control of large, established tumor xenografts with genetically retargeted human T cells containing CD28 and CD137 domains. *Proc. Natl. Acad. Sci. U. S. A.* **106**, 3360-3365, (2009).

8 Ramos, C. A. *et al.* *In* *vivo* fate and activity of second- versus third-generation CD19-specific CAR-T cells in B cell non-Hodgkin's lymphomas. *Mol. Ther.* **26**, 2727-2737, (2018).

9 Guedan, S. *et al.* Enhancing CAR T cell persistence through ICOS and 4-1BB costimulation. *JCI Insight* **3**, e96976, (2018).

10 Porter, D. L. Chimeric antigen receptor-modified T cells in chronic lymphoid leukemia. *N. Engl. J. Med.* **365**, 725-733, (2011).

11 Schuster, S. J. *et al.* Chimeric antigen receptor T cells in refractory B-cell lymphomas. *N. Engl. J. Med.* **377**, 2545-2554, (2017).

12 Maude, S. L. *et al.* Tisagenlecleucel in children and young adults with B-Cell lymphoblastic leukemia. *N. Engl. J. Med.* **378**, 439-448, (2018).

13 Schuster, S. J. *et al.* Tisagenlecleucel in adult relapsed or refractory diffuse large B-cell lymphoma. *N. Engl. J. Med.* **380**, 45-56, (2019).

14 Mullard, A. FDA approves first CAR T therapy. *Nat. Rev. Drug Discov.* **16**, 669, (2017).

15 Tran, E., Longo, D. L. & Urba, W. J. A milestone for CAR T cells. *N. Engl. J. Med.* **377**, 2593-2596, (2017).

16 Prasad, V. Immunotherapy: Tisagenlecleucel - the first approved CAR-T-cell therapy: implications for payers and policy makers. *Nat. Rev. Clin. Oncol.* **15**, 11-12, (2018).

17 Neelapu, S. S. *et al.* Axicabtagene ciloleucel CAR T-cell therapy in refractory large B-cell lymphoma. *N. Engl. J. Med.* **377**, 2531-2544, (2017).

18 Mullard, A. Second anticancer CAR T therapy receives FDA approval. *Nat. Rev. Drug Discov.* **16**, 818, (2017).

19 Wang, M. *et al.* KTE-X19 CAR T-cell therapy in relapsed or refractory mantle-cell lymphoma. *N. Engl. J. Med.* **382**, 1331-1342, (2020).

20 FDA okays second CAR-T for Kite. *Nat. Biotechnol.* **38**, 1012, (2020).

21 Abramson, J. S. *et al.* Lisocabtagene maraleucel for patients with relapsed or refractory large B-cell lymphomas (TRANSCEND NHL 001): a multicentre seamless design study. *Lancet* **396**, 839-852, (2020).

22 Mullard, A. FDA approves fourth CAR-T cell therapy. *Nat. Rev. Drug Discov.* **20**, 166, (2021).

23 Raje, N. *et al.* Anti-BCMA CAR T-cell therapy bb2121 in relapsed or refractory multiple myeloma. *N. Engl. J. Med.* **380**, 1726-1737, (2019).

24 Munshi, N. C. *et al.* Idecabtagene vicleucel in relapsed and refractory multiple myeloma. *N. Engl. J. Med.* **384**, 705-716, (2021).

25 Mullard, A. FDA approves first BCMA-targeted CAR-T cell therapy. *Nat. Rev. Drug Discov.* **20**, 332, (2021).

26 First CAR-T therapy to target BCMA gets FDA nod. *Nat. Biotechnol.* **39**, 531, (2021).

27 Berdeja, J. G. *et al.* Ciltacabtagene autoleucel, a B-cell maturation antigen-directed chimeric antigen receptor T-cell therapy in patients with relapsed or refractory multiple myeloma (CARTITUDE-1): a phase 1b/2 open-label study. *Lancet* **398**, 314-324, (2021).

28 Mi, J. Q. *et al.* Phase II, open-label study of ciltacabtagene autoleucel, an anti-B-cell maturation antigen chimeric antigen receptor-T-cell therapy, in Chinese patients with relapsed/refractory multiple myeloma (CARTIFAN-1). *J. Clin. Oncol.* **41**, 1275-1284, (2023).

29 Mullard, A. FDA approves second BCMA-targeted CAR-T cell therapy. *Nat. Rev. Drug Discov.* **21**, 249, (2022).

30 Zhang, C., Liu, J., Zhong, J. F. & Zhang, X. Engineering CAR-T cells. *Biomark. Res.* **5**, 22, (2017).

31 Hegde, R. S. & Bernstein, H. D. The surprising complexity of signal sequences. *Trends Biochem. Sci.* **31**, 563-571, (2006).

32 Hegde, R. S. & Kang, S. W. The concept of translocational regulation. *J. Cell Biol.* **182**, 225-232, (2008).

33 Nyathi, Y., Wilkinson, B. M. & Pool, M. R. Co-translational targeting and translocation of proteins to the endoplasmic reticulum. *Biochim. Biophys. Acta* **1833**, 2392-2402, (2013).

34 Hegde, R. S. & Keenan, R. J. The mechanisms of integral membrane protein biogenesis. *Nat. Rev. Mol. Cell Biol.* **23**, 107-124, (2022).

35 Li, F. *et al.* A study on influence of different signal peptides on anti-tumor effect of chimeric antigen receptor (CAR) T cells *China Oncology* **32**, 142-151, (2022).

36 Ping, Y. *et al.* Augmenting the effectiveness of CAR-T cells by enhanced self-delivery of PD-1-neutralizing scFv. *Front Cell Dev Biol* **8**, 803, (2020).

37 Krause, A. *et al.* Antigen-dependent CD28 signaling selectively enhances survival and proliferation in genetically modified activated human primary T lymphocytes. *J. Exp. Med.* **188**, 619-626, (1998).

38 Brentjens, R. J. *et al.* Eradication of systemic B-cell tumors by genetically targeted human T lymphocytes co-stimulated by CD80 and interleukin-15. *Nat. Med.* **9**, 279-286, (2003).

39 Imai, C. *et al.* Chimeric receptors with 4-1BB signaling capacity provoke potent cytotoxicity against acute lymphoblastic leukemia. *Leukemia* **18**, 676-684, (2004).

40 Song, D. G. *et al.* In vivo persistence, tumor localization, and antitumor activity of CAR-engineered T cells is enhanced by costimulatory signaling through CD137 (4-1BB). *Cancer Res.* **71**, 4617-4627, (2011).

41 Fedorov, V. D., Themeli, M. & Sadelain, M. PD-1- and CTLA-4-based inhibitory chimeric antigen receptors (iCARs) divert off-target immunotherapy responses. *Sci. Transl. Med.* **5**, 215ra172, (2013).

42 Kloss, C. C., Condomines, M., Cartellieri, M., Bachmann, M. & Sadelain, M. Combinatorial antigen recognition with balanced signaling promotes selective tumor eradication by engineered T cells. *Nat. Biotechnol.* **31**, 71-75, (2013).

43 Ellebrecht, C. T. *et al.* Reengineering chimeric antigen receptor T cells for targeted therapy of autoimmune disease. *Science* **353**, 179-184, (2016).

44 Rodgers, D. T. *et al.* Switch-mediated activation and retargeting of CAR-T cells for B-cell malignancies. *Proc. Natl. Acad. Sci. U. S. A.* **113**, E459-E468, (2016).

45 Eyquem, J. *et al.* Targeting a CAR to the TRAC locus with CRISPR/Cas9 enhances tumour rejection. *Nature* **543**, 113-117, (2017).

46 Choi, B. D. *et al.* CAR-T cells secreting BiTEs circumvent antigen escape without detectable toxicity. *Nat. Biotechnol.* **37**, 1049-1058, (2019).

47 Feucht, J. *et al.* Calibration of CAR activation potential directs alternative T cell fates and therapeutic potency. *Nat. Med.* **25**, 82-88, (2019).

48 Ma, L. *et al.* Enhanced CAR-T cell activity against solid tumors by vaccine boosting through the chimeric receptor. *Science* **365**, 162-168, (2019).

49 Raj, D. *et al.* Switchable CAR-T cells mediate remission in metastatic pancreatic ductal adenocarcinoma. *Gut* **68**, 1052-1064, (2019).

50 Amor, C. *et al.* Senolytic CAR T cells reverse senescence-associated pathologies. *Nature* **583**, 127-132, (2020).

51 Giordano-Attianese, G. *et al.* A computationally designed chimeric antigen receptor provides a small-molecule safety switch for T-cell therapy. *Nat. Biotechnol.* **38**, 426-432, (2020).

52 Nguyen, N. T. *et al.* Nano-optogenetic engineering of CAR T cells for precision immunotherapy with enhanced safety. *Nat. Nanotechnol.* **16**, 1424-1434, (2021).

53 Xie, G. *et al.* CAR-T cells targeting a nucleophosmin neoepitope exhibit potent specific activity in mouse models of acute myeloid leukaemia. *Nat. Biomed. Eng.* **5**, 399-413, (2021).

54 Zhang, A. Q. *et al.* Universal redirection of CAR T cells against solid tumours via membrane-inserted ligands for the CAR. *Nat. Biomed. Eng.* **7**, 1113-1128, (2023).

55 Zhong, X. S., Matsushita, M., Plotkin, J., Riviere, I. & Sadelain, M. Chimeric antigen receptors combining 4-1BB and CD28 signaling domains augment PI3kinase/AKT/Bcl-XL activation and CD8+ T cell-mediated tumor eradication. *Mol. Ther.* **18**, 413-420, (2010).

56 Imai, C., Iwamoto, S. & Campana, D. Genetic modification of primary natural killer cells overcomes inhibitory signals and induces specific killing of leukemic cells. *Blood* **106**, 376-383, (2005).

57 Li, Y., Hermanson, D. L., Moriarity, B. S. & Kaufman, D. S. Human iPSC-derived natural killer cells engineered with chimeric antigen receptors enhance anti-tumor activity. *Cell Stem Cell* **23**, 181-192.e5, (2018).

58 Yu, M. *et al.* Development of GPC3-specific chimeric antigen receptor-engineered natural killer cells for the treatment of hepatocellular carcinoma. *Mol. Ther.* **26**, 366-378, (2018).

59 Morrissey, M. A. *et al.* Chimeric antigen receptors that trigger phagocytosis. *Elife* **7**, (2018).

60 Klichinsky, M. *et al.* Human chimeric antigen receptor macrophages for cancer immunotherapy. *Nat. Biotechnol.* **38**, 947-953, (2020).

61 Chen, C. *et al.* Intracavity generation of glioma stem cell-specific CAR macrophages primes locoregional immunity for postoperative glioblastoma therapy. *Sci. Transl. Med.* **14**, eabn1128, (2022).

62 Wilkie, S. *et al.* Dual targeting of ErbB2 and MUC1 in breast cancer using chimeric antigen receptors engineered to provide complementary signaling. *J. Clin. Immunol.* **32**, 1059-1070, (2012).

63 Haso, W. *et al.* Anti-CD22-chimeric antigen receptors targeting B-cell precursor acute lymphoblastic leukemia. *Blood* **121**, 1165-1174, (2013).

64 Jonnalagadda, M. *et al.* Chimeric antigen receptors with mutated IgG4 Fc spacer avoid fc receptor binding and improve T cell persistence and antitumor efficacy. *Mol. Ther.* **23**, 757-768, (2015).

65 Schneider, D. *et al.* A tandem CD19/CD20 CAR lentiviral vector drives on-target and off-target antigen modulation in leukemia cell lines. *J. Immunother. Cancer* **5**, 42, (2017).

66 Shah, N. N. *et al.* Bispecific anti-CD20, anti-CD19 CAR T cells for relapsed B cell malignancies: a phase 1 dose escalation and expansion trial. *Nat. Med.* **26**, 1569-1575, (2020).

67 Wu, W. *et al.* Multiple signaling roles of CD3ε and its application in CAR-T cell therapy. *Cell* **182**, 855-871.e23, (2020).

68 Kloess, S. *et al.* Preclinical assessment of suitable natural killer cell sources for chimeric antigen receptor natural killer-based “off-the-shelf" acute myeloid leukemia immunotherapies. *Hum. Gene Ther.* **30**, 381-401, (2019).

69 Oberschmidt, O. *et al.* Development of automated separation, expansion, and quality control protocols for clinical-scale manufacturing of primary human NK cells and alpharetroviral chimeric antigen receptor engineering. *Hum. Gene Ther. Methods* **30**, 102-120, (2019).

70 Heitzeneder, S. *et al.* GPC2-CAR T cells tuned for low antigen density mediate potent activity against neuroblastoma without toxicity. *Cancer Cell* **40**, 53-69.e9, (2022).

71 Tousley, A. M. *et al.* Co-opting signalling molecules enables logic-gated control of CAR T cells. *Nature* **615**, 507-516, (2023).

72 Ahmed, N. *et al.* HER2-specific T cells target primary glioblastoma stem cells and induce regression of autologous experimental tumors. *Clin. Cancer Res.* **16**, 474-485, (2010).

73 Long, A. H. *et al.* 4-1BB costimulation ameliorates T cell exhaustion induced by tonic signaling of chimeric antigen receptors. *Nat. Med.* **21**, 581-590, (2015).

74 Schonfeld, K. *et al.* Selective inhibition of tumor growth by clonal NK cells expressing an ErbB2/HER2-specific chimeric antigen receptor. *Mol. Ther.* **23**, 330-338, (2015).

75 Schnalzger, T. E. *et al.* 3D model for CAR-mediated cytotoxicity using patient-derived colorectal cancer organoids. *EMBO J.* **38**, (2019).

76 Moot, R. *et al.* Genetic engineering of chimeric antigen receptors using lamprey derived variable lymphocyte receptors. *Mol. Ther. Oncolytics* **3**, 16026, (2016).

77 Ghosh, A. *et al.* Donor CD19 CAR T cells exert potent graft-versus-lymphoma activity with diminished graft-versus-host activity. *Nat. Med.* **23**, 242-249, (2017).

78 Eshhar, Z., Waks, T., Gross, G. & Schindler, D. G. Specific activation and targeting of cytotoxic lymphocytes through chimeric single chains consisting of antibody-binding domains and the γ or ζ subunits of the immunoglobulin and T-cell receptors. *Proc. Natl. Acad. Sci. U. S. A.* **90**, 720-724, (1993).

79 Chmielewski, M. & Abken, H. CAR T cells releasing IL-18 convert to T-bet(high) FoxO1(low) effectors that exhibit augmented activity against advanced solid tumors. *Cell Rep.* **21**, 3205-3219, (2017).

80 Till, B. G. *et al.* Adoptive immunotherapy for indolent non-Hodgkin lymphoma and mantle cell lymphoma using genetically modified autologous CD20-specific T cells. *Blood* **112**, 2261-2271, (2008).

81 Muller, N. *et al.* Engineering NK cells modified with an EGFRvIII-specific chimeric antigen receptor to overexpress CXCR4 improves immunotherapy of CXCL12/SDF-1α-secreting glioblastoma. *J. Immunother.* **38**, 197-210, (2015).

82 Zhang, W. *et al.* Chimeric antigen receptor macrophage therapy for breast tumours mediated by targeting the tumour extracellular matrix. *Br. J. Cancer* **121**, 837-845, (2019).

83 Labanieh, L., Majzner, R. G. & Mackall, C. L. Programming CAR-T cells to kill cancer. *Nat. Biomed. Eng.* **2**, 377-391, (2018).

84 Rafiq, S., Hackett, C. S. & Brentjens, R. J. Engineering strategies to overcome the current roadblocks in CAR T cell therapy. *Nat. Rev. Clin. Oncol.* **17**, 147-167, (2020).

85 Hombach, A., Hombach, A. A. & Abken, H. Adoptive immunotherapy with genetically engineered T cells: modification of the IgG1 Fc 'spacer' domain in the extracellular moiety of chimeric antigen receptors avoids 'off-target' activation and unintended initiation of an innate immune response. *Gene Ther.* **17**, 1206-1213, (2010).

86 Hudecek, M. *et al.* Receptor affinity and extracellular domain modifications affect tumor recognition by ROR1-specific chimeric antigen receptor T cells. *Clin. Cancer Res.* **19**, 3153-3164, (2013).

87 Hudecek, M. *et al.* The nonsignaling extracellular spacer domain of chimeric antigen receptors is decisive for in vivo antitumor activity. *Cancer Immunol. Res.* **3**, 125-135, (2015).

88 Qin, L. *et al.* Incorporation of a hinge domain improves the expansion of chimeric antigen receptor T cells. *J. Hematol. Oncol.* **10**, 68, (2017).

89 Alabanza, L. *et al.* Function of novel anti-CD19 chimeric antigen receptors with human variable regions is affected by hinge and transmembrane domains. *Mol. Ther.* **25**, 2452-2465, (2017).

90 Ying, Z. *et al.* A safe and potent anti-CD19 CAR T cell therapy. *Nat. Med.* **25**, 947-953, (2019).

91 Li, N. *et al.* The IgG4 hinge with CD28 transmembrane domain improves V(H)H-based CAR T cells targeting a membrane-distal epitope of GPC1 in pancreatic cancer. *Nat. Commun.* **14**, 1986, (2023).

92 Chen, X., Zaro, J. L. & Shen, W. C. Fusion protein linkers: property, design and functionality. *Adv. Drug Deliv. Rev.* **65**, 1357-1369, (2013).

93 Yusakul, G., Sakamoto, S., Pongkitwitoon, B., Tanaka, H. & Morimoto, S. Effect of linker length between variable domains of single chain variable fragment antibody against daidzin on its reactivity. *Biosci. Biotechnol. Biochem.* **80**, 1306-1312, (2016).

94 Dolezal, O. *et al.* ScFv multimers of the anti-neuraminidase antibody NC10: shortening of the linker in single-chain Fv fragment assembled in V(L) to V(H) orientation drives the formation of dimers, trimers, tetramers and higher molecular mass multimers. *Protein Eng.* **13**, 565-574, (2000).

95 Richman, S. A. *et al.* High-affinity GD2-specific CAR T cells induce fatal encephalitis in a preclinical neuroblastoma model. *Cancer Immunol. Res.* **6**, 36-46, (2018).

96 Fujiwara, K., Masutani, M., Tachibana, M. & Okada, N. Impact of scFv structure in chimeric antigen receptor on receptor expression efficiency and antigen recognition properties. *Biochem. Biophys. Res. Commun.* **527**, 350-357, (2020).

97 Singh, N. *et al.* Antigen-independent activation enhances the efficacy of 4-1BB-costimulated CD22 CAR T cells. *Nat. Med.* **27**, 842-850, (2021).

98 Fry, T. J. *et al.* CD22-targeted CAR T cells induce remission in B-ALL that is naive or resistant to CD19-targeted CAR immunotherapy. *Nat. Med.* **24**, 20-28, (2018).

99 Shah, N. N. *et al.* CD4/CD8 T-cell selection affects chimeric antigen receptor (CAR) T-cell potency and toxicity: updated results from a phase I anti-CD22 CAR T-cell trial. *J. Clin. Oncol.* **38**, 1938-1950, (2020).

100 Liu, D. & Zhao, J. Cytokine release syndrome: grading, modeling, and new therapy. *J. Hematol. Oncol.* **11**, 121, (2018).

101 Morris, E. C., Neelapu, S. S., Giavridis, T. & Sadelain, M. Cytokine release syndrome and associated neurotoxicity in cancer immunotherapy. *Nat. Rev. Immunol.* **22**, 85-96, (2022).

102 Liu, D., Zhao, J. & Song, Y. Engineering switchable and programmable universal CARs for CAR T therapy. *J. Hematol. Oncol.* **12**, 69, (2019).

103 Cho, J. H., Collins, J. J. & Wong, W. W. Universal chimeric antigen receptors for multiplexed and logical control of T cell responses. *Cell* **173**, 1426-1438.e11, (2018).

104 Cho, J. H. *et al.* Engineering advanced logic and distributed computing in human CAR immune cells. *Nat. Commun.* **12**, 792, (2021).

105 Urbanska, K. *et al.* A universal strategy for adoptive immunotherapy of cancer through use of a novel T-cell antigen receptor. *Cancer Res.* **72**, 1844-1852, (2012).

106 Seitz, C. M. *et al.* Novel adapter CAR-T cell technology for precisely controllable multiplex cancer targeting. *Oncoimmunology* **10**, 2003532, (2021).

107 Nixdorf, D. *et al.* Adapter CAR T cells to counteract T-cell exhaustion and enable flexible targeting in AML. *Leukemia* **37**, 1298-1310, (2023).

108 Tamada, K. *et al.* Redirecting gene-modified T cells toward various cancer types using tagged antibodies. *Clin. Cancer Res.* **18**, 6436-6445, (2012).

109 Kim, M. S. *et al.* Redirection of genetically engineered CAR-T cells using bifunctional small molecules. *J. Am. Chem. Soc.* **137**, 2832-2835, (2015).

110 Ma, J. S. *et al.* Versatile strategy for controlling the specificity and activity of engineered T cells. *Proc. Natl. Acad. Sci. U. S. A.* **113**, E450-E458, (2016).

111 Lee, Y. G. *et al.* Use of a single CAR T cell and several bispecific adapters facilitates eradication of multiple antigenically different solid tumors. *Cancer Res.* **79**, 387-396, (2019).

112 Ruffo, E. *et al.* Post-translational covalent assembly of CAR and synNotch receptors for programmable antigen targeting. *Nat. Commun.* **14**, 2463, (2023).

113 Herzig, E. *et al.* Attacking latent HIV with *convertible*CAR-T cells, a highly adaptable killing platform. *Cell* **179**, 880-894.e10, (2019).

114 Lajoie, M. J. *et al.* Designed protein logic to target cells with precise combinations of surface antigens. *Science* **369**, 1637-1643, (2020).

115 Qi, J. *et al.* Chemically programmable and switchable CAR-T therapy. *Angew. Chem. Int. Ed. Engl.* **59**, 12178-12185, (2020).

116 Hong, M., Clubb, J. D. & Chen, Y. Y. Engineering CAR-T cells for next-generation cancer therapy. *Cancer Cell* **38**, 473-488, (2020).

117 Lee, S., Khalil, A. S. & Wong, W. W. Recent progress of gene circuit designs in immune cell therapies. *Cell Syst.* **13**, 864-873, (2022).

118 Chen, Y. Y. Increasing T cell versatility with SUPRA CARs. *Cell* **173**, 1316-1317, (2018).

119 Zhao, J., Lin, Q., Song, Y. & Liu, D. Universal CARs, universal T cells, and universal CAR T cells. *J. Hematol. Oncol.* **11**, 132, (2018).

120 Sadelain, M., Brentjens, R. & Riviere, I. The basic principles of chimeric antigen receptor design. *Cancer Discov.* **3**, 388-398, (2013).

121 Jayaraman, J. *et al.* CAR-T design: elements and their synergistic function. *EBioMedicine* **58**, 102931, (2020).

122 Haynes, N. M. *et al.* Redirecting mouse CTL against colon carcinoma: superior signaling efficacy of single-chain variable domain chimeras containing TCR-ζ vs FcεRI-γ. *J. Immunol.* **166**, 182-187, (2001).

123 Heuser, C., Hombach, A., Losch, C., Manista, K. & Abken, H. T-cell activation by recombinant immunoreceptors: impact of the intracellular signalling domain on the stability of receptor expression and antigen-specific activation of grafted T cells. *Gene Ther.* **10**, 1408-1419, (2003).

124 Zhao, Y. *et al.* A herceptin-based chimeric antigen receptor with modified signaling domains leads to enhanced survival of transduced T lymphocytes and antitumor activity. *J. Immunol.* **183**, 5563-5574, (2009).

125 Kochenderfer, J. N., Yu, Z., Frasheri, D., Restifo, N. P. & Rosenberg, S. A. Adoptive transfer of syngeneic T cells transduced with a chimeric antigen receptor that recognizes murine CD19 can eradicate lymphoma and normal B cells. *Blood* **116**, 3875-3886, (2010).

126 Majzner, R. G. *et al.* Tuning the antigen density requirement for CAR T-cell activity. *Cancer Discov.* **10**, 702-723, (2020).

127 James, J. R. Tuning ITAM multiplicity on T cell receptors can control potency and selectivity to ligand density. *Sci. Signal.* **11**, eaan1088, (2018).

128 Cappell, K. M. & Kochenderfer, J. N. A comparison of chimeric antigen receptors containing CD28 versus 4-1BB costimulatory domains. *Nat. Rev. Clin. Oncol.* **18**, 715-727, (2021).

129 Finney, H. M., Lawson, A. D., Bebbington, C. R. & Weir, A. N. Chimeric receptors providing both primary and costimulatory signaling in T cells from a single gene product. *J. Immunol.* **161**, 2791-2797, (1998).

130 Haynes, N. M. *et al.* Single-chain antigen recognition receptors that costimulate potent rejection of established experimental tumors. *Blood* **100**, 3155-3163, (2002).

131 Maher, J., Brentjens, R. J., Gunset, G., Riviere, I. & Sadelain, M. Human T-lymphocyte cytotoxicity and proliferation directed by a single chimeric TCRζ /CD28 receptor. *Nat. Biotechnol.* **20**, 70-75, (2002).

132 Kowolik, C. M. *et al.* CD28 costimulation provided through a CD19-specific chimeric antigen receptor enhances *in vivo* persistence and antitumor efficacy of adoptively transferred T cells. *Cancer Res.* **66**, 10995-11004, (2006).

133 Brentjens, R. J. *et al.* Genetically targeted T cells eradicate systemic acute lymphoblastic leukemia xenografts. *Clin. Cancer Res.* **13**, 5426-5435, (2007).

134 Kawalekar, O. U. *et al.* Distinct signaling of coreceptors regulates specific metabolism pathways and impacts memory development in CAR T cells. *Immunity* **44**, 380-390, (2016).

135 Salter, A. I. *et al.* Phosphoproteomic analysis of chimeric antigen receptor signaling reveals kinetic and quantitative differences that affect cell function. *Sci. Signal.* **11**, (2018).

136 Ying, Z. *et al.* Parallel comparison of 4-1BB or CD28 co-stimulated CD19-targeted CAR-T cells for B cell non-Hodgkin's lymphoma. *Mol. Ther. Oncolytics* **15**, 60-68, (2019).

137 Finney, H. M., Akbar, A. N. & Lawson, A. D. Activation of resting human primary T cells with chimeric receptors: costimulation from CD28, inducible costimulator, CD134, and CD137 in series with signals from the TCRζ chain. *J. Immunol.* **172**, 104-113, (2004).

138 Shen, C. J. *et al.* Chimeric antigen receptor containing ICOS signaling domain mediates specific and efficient antitumor effect of T cells against EGFRvIII expressing glioma. *J. Hematol. Oncol.* **6**, 33, (2013).

139 Guedan, S. *et al.* ICOS-based chimeric antigen receptors program bipolar TH17/TH1 cells. *Blood* **124**, 1070-1080, (2014).

140 Pule, M. A. *et al.* A chimeric T cell antigen receptor that augments cytokine release and supports clonal expansion of primary human T cells. *Mol. Ther.* **12**, 933-941, (2005).

141 Shaffer, D. R. *et al.* T cells redirected against CD70 for the immunotherapy of CD70-positive malignancies. *Blood* **117**, 4304-4314, (2011).

142 Song, D. G. *et al.* CD27 costimulation augments the survival and antitumor activity of redirected human T cells in vivo. *Blood* **119**, 696-706, (2012).

143 Mata, M. *et al.* Inducible activation of MyD88 and CD40 in CAR T cells results in controllable and potent antitumor activity in preclinical solid tumor models. *Cancer Discov.* **7**, 1306-1319, (2017).

144 Barber, A. & Sentman, C. L. Chimeric NKG2D T cells require both T cell- and host-derived cytokine secretion and perforin expression to increase tumor antigen presentation and systemic immunity. *J. Immunol.* **183**, 2365-2372, (2009).

145 Song, D. G. *et al.* Chimeric NKG2D CAR-expressing T cell-mediated attack of human ovarian cancer is enhanced by histone deacetylase inhibition. *Hum. Gene Ther.* **24**, 295-305, (2013).

146 Zhang, T. & Sentman, C. L. Mouse tumor vasculature expresses NKG2D ligands and can be targeted by chimeric NKG2D-modified T cells. *J. Immunol.* **190**, 2455-2463, (2013).

147 van der Stegen, S. J., Hamieh, M. & Sadelain, M. The pharmacology of second-generation chimeric antigen receptors. *Nat. Rev. Drug Discov.* **14**, 499-509, (2015).
